# Characterizing and Predicting nvPM Size Distributions for Aviation Emission Inventories and Environmental Impact

**DOI:** 10.1021/acs.est.4c02538

**Published:** 2024-06-10

**Authors:** Lukas Durdina, Eliot Durand, Jacinta Edebeli, Curdin Spirig, Benjamin T. Brem, Miriam Elser, Frithjof Siegerist, Mark Johnson, Yura A. Sevcenco, Andrew P. Crayford

**Affiliations:** † Centre for Aviation, ZHAW Zurich University of Applied Sciences, Winterthur CH-8401, Switzerland; ‡ Cardiff School of Engineering, Cardiff University, Wales CF24 3AA, U.K.; § Laboratory for Atmospheric Chemistry, Paul Scherrer Institute, Villigen CH-5232, Switzerland; ∥ Laboratory for Automotive Powertrain Technologies, Empa, Dübendorf CH-8600, Switzerland; ⊥ SR Technics Switzerland AG, Zurich-Airport,Kloten CH-8058, Switzerland; # Rolls-Royce,Plc, Sin A-37 PO Box 31, Derby DE24 8BJ, U.K.

**Keywords:** aviation, non-CO_2_ emissions, air pollution, particulate matter, nvPM, particle size distribution

## Abstract

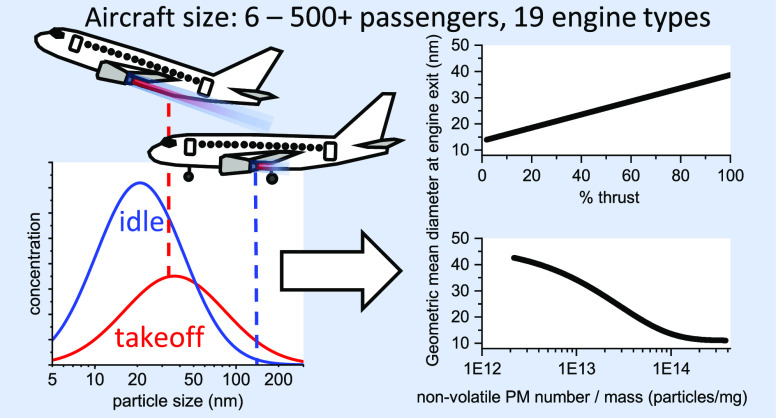

Concerns about civil aviation’s air quality and environmental impacts have led to recent regulations on nonvolatile particulate matter (nvPM) mass and number emissions. Although these regulations do not mandate measuring particle size distribution (PSD), understanding PSDs is vital for assessing the environmental impacts of aviation nvPM. This study introduces a comprehensive data set detailing PSD characteristics of 42 engines across 19 turbofan types, ranging from unregulated small business jets to regulated large commercial aircraft. Emission tests were independently performed by using the European and Swiss reference nvPM sampling and measurement systems with parallel PSD measurements. The geometric mean diameter (GMD) at the engine exit strongly correlated with the nvPM number-to-mass ratio (N/M) and thrust, varying from 7 to 52 nm. The engine-exit geometric standard deviation ranged from 1.7 to 2.5 (mean of 2.05). The study proposes empirical correlations to predict GMD from N/M data of emissions-certified engines. These predictions are expected to be effective for conventional rich-burn engines and might be extended to novel combustor technologies if additional data become available. The findings support the refinement of emission models and help in assessing the aviation non-CO_2_ climate and air quality impacts.

## Abstract 2

Particle size distributions measured in the exhausts of a wide range of aircraft engines were used to develop parametrizations to improve assessments of aviation’s air quality and climate impacts.

## Introduction

The rapid expansion of global aviation has brought about significant technological advancements but also raised concerns about climate impacts and local air quality. Central to these concerns is the issue of nonvolatile particulate matter (nvPM) emissions from aircraft engines, which impact atmospheric chemistry, radiative forcing, and human health.^1−3^ A key aspect of understanding nvPM’s environmental impact is its particle size distribution (PSD). The PSD is critical because it determines the particles’ residence time in the atmosphere, their health effects, their interaction with solar radiation, and their potential to form contrail and cloud condensation nuclei, one of the most uncertain aspects of aviation’s climate impacts.^4,5^

Historically, PSD measurements in aircraft engine exhaust were used to estimate nvPM emission indices (EI, amount of pollutant per kg fuel burned) at ground level and cruising altitudes.^6−8^ Electrical mobility-based sizing instruments have reported high number concentrations of ultrafine particles in exhaust plumes, challenging the adequacy of the traditional smoke number (SN) standard, which focused primarily on visibility impacts.^9^ These instruments, operating within complex sampling and measurement systems with long sampling lines, face significant particle losses and alterations to the PSD between the engine exit plane (EEP) and the instrument.^10^ Such complexities, along with the size-dependent nature of particle loss, underscore the importance of accurate PSD measurements for environmental and health risk assessments.

Studies utilizing various sampling system designs and mobility-based sizing instruments have consistently found the nonvolatile aerosol fraction in rich-burn aircraft engine exhaust to be typically log-normally distributed, with geometric mean diameters (GMDs) ranging from 15 to 50 nm and geometric standard deviations (GSDs) from 1.5 to 2.3.^11−23^ These parameters, critical for predicting nvPM impacts, are either direct instrument readings or inconsistently corrected for particle losses to the EEP, highlighting the challenges in obtaining accurate PSD data representative of the engine exit.

PSD measurements have provided mounting evidence of high number concentrations of nanoparticles in aircraft engine exhaust, which has precipitated the development and introduction of global nvPM emission standards. The International Civil Aviation Organization (ICAO) nvPM standards apply to all civil turbofan and turbojet engines with a rated thrust >26.7 kN (6000 lb). Specifically, the CAEP/10 (10th cycle of the Committee on Aviation Environmental Protection) nvPM mass concentration standard, introduced in 2020, directly replaces SN by addressing the exhaust nonvisibility criterion. Following this, the CAEP/11 nvPM Landing and Takeoff (LTO) mass and number standard, introduced in 2023, regulates the nvPM mass and number emissions from the reference LTO cycle, intended to represent peak traffic operations below 3000 ft, where pollutants can detrimentally impact local air quality.^9,24−26^ The certified nvPM mass and number LTO emissions are reported in the ICAO Aircraft Engine Emissions Databank (EEDB).^27^ However, current regulations do not mandate PSD measurements for nvPM certification due to uncertainties and challenges in defining and traceably measuring particle sizes in the ultrafine range.

Significant particle losses in the nvPM sampling systems, up to 90% for the smallest particles (around 10 nm), necessitate a system loss correction to estimate the emissions released into the environment accurately.^10,28,29^ The prescribed nvPM system loss correction methodology in the ICAO Annex 16 Vol. II uses standardized nvPM mass and number measurements and assumes a monomodal log-normal PSD at the EEP with a GSD of 1.8 and unit particle effective density (1 g/cm^3^).^9^

Assumptions about aircraft engine nvPM PSDs are also integral to models that convert mass-based emissions to number-based emissions for older engine types and small engines not certified for nvPM. For such engines, SN can be used for estimating nvPM mass and number EIs using methods like the First Order Approximation (FOA 4.0) or SCOPE11.^30,31^ Yet, these estimations again depend on assumed GMD and GSD values for the nvPM number EI at the EEP.

PSD properties are needed in models predicting engine emissions at cruising altitudes and contrail formation studies.^32,33^ The models use GMD, GSD, and density assumptions to convert nvPM mass to number EIs, which influence contrail properties and their projected climate impact.^34,35^ The nvPM GMD also plays a role in contrail microphysics through its influence on particle activation processes. The activation efficiency of soot particles increases with size because of the increased surface area, facilitating condensation and ice nucleation.^36,37^

Despite extensive studies, a gap remains in using standardized nvPM sampling and measurement systems across different engine types and conditions, with parallel PSD measurement corrected for particle losses and representative of the EEP. Our research addresses this gap by compiling an extensive data set using European (EUR) and Swiss (CH) reference nvPM sampling and measurement systems during full-scale engine tests. The measured PSDs were corrected for size-dependent system loss to provide GMD and GSD characteristics representative of nvPM emitted into the atmosphere. This study correlates these PSD properties with regulatory nvPM number, mass emissions, and engine thrust. It also reports an average nvPM effective density derived using the PSD volume and measured nvPM mass. Our findings provide PSD characteristics representative of in-service aircraft engines across LTO operations, offering insights for refinement of the conversion from mass-based to number-based emissions, and can inform local air quality studies and predictive models for nvPM emissions at cruising altitudes and contrail formation.

## Materials and Methods

### Engine Emission Tests

Over 7 years, emission tests of 42 commercial turbofan engines across 19 distinct types and 9 manufacturers, covering thrust ratings from 15 to 350 kN, were conducted in static sea level test cells and during static tests with engines mounted on aircraft. Notably, these engines had various rich-burn combustion systems, and they did not include staged or premixed lean-burn combustion systems, as featured in some engine models by CFM International and General Electric.^38^

Of the engines tested, 17 underwent dedicated emission tests, whereas the remaining 25 were evaluated for emissions during pass-off performance tests post repair or overhaul. The latter adhered to the engine service manuals, including typically five to seven thrust levels from idle to takeoff. The dedicated tests focused on the regulatory LTO cycle, and a test matrix typically consisted of 8–15 test points from idle to takeoff thrust. For the test cell measurements, net thrust was determined from a correlation between the combustor inlet temperature T3 and static thrust at standard sea level (15 °C, 101.325 kPa), in line with the ICAO emissions certification standard.^9^ Where engines of the same type had varied rated takeoff thrusts, they were normalized to the maximum rated thrust for the sake of consistency. For aircraft-mounted engines, thrust estimations were based on correlations with engine speed (N1, low-pressure shaft speed) at the standard sea level. All engines burned Jet A-1 fuel without synthetic blending components.

### Exhaust Sampling and nvPM Measurement

Exhaust sampling employed either multihole or traversable single-hole probes at 0.1–1.7 m downstream of the EEP, following the SAE Aerospace Recommended Practice (ARP) 6320^39^ and the ICAO Annex 16 standard^9^ sampling and measurement protocols. Detailed descriptions of the EUR and CH systems are available in the existing literature.^14,17,18,25^ Each system reports mass and number concentrations from nominally identical nvPM instruments: the AVL Micro Soot Sensor (MSS) for the nvPM mass and the AVL Advanced Particle Counter (APC) for the nvPM number concentration. The EUR and CH systems were compared in parallel on large turbofan engines during the nvPM standard development.^14^ Recent comparisons of these systems using a rich-burn/quick-quench/lean-burn (RQL) combustor rig with varied jet fuel blends highlighted excellent agreement of emission indices after joint calibration: less than 3% for nvPM mass and under 1% for nvPM number across all conditions and fuels.^40^

### Particle Size Distribution Measurement

The EUR system used a Cambustion DMS500 fast particle size spectrometer (10 Hz) for PSD measurements from 5 to 1000 nm processed using the monomodal aggregate inversion matrix generated using mini-CAST soot. In contrast, the CH system utilized a TSI Scanning Mobility Particle Sizer (SMPS) Model 3938 equipped with an electrostatic classifier Model 3082, a bipolar Kr-85 aerosol neutralizer Model 3077A, a long Differential Mobility Analyzer (DMA) Model 3081, and a condensation particle counter (CPC) Model 3776. This fast-scanning SMPS conducted scans in 18 and 30 s, capturing sizes from 7 to 240 nm. The scanning times were appropriate for the high-concentration polydisperse aerosol without any measurable effect on the sizing accuracy, GSD, and total concentration compared to 60s scans.^41^ Both instruments sampled diluted exhaust in parallel with nvPM mass and number instruments.^17,18^ Comparisons on the aforementioned combustor rig showed that both instruments agreed within a 5% margin for GMD and GSD.^10,40^ Notably, this study did not include a catalytic stripper (CS) for removing volatile compounds upstream of the size analyzers. The standardized nvPM sampling system with heated lines and rapid dilution suppresses the formation of volatile PM when the system is operated correctly and without any unburned fuel or oil contamination. This decision was validated in selected tests with and without a CS, showing consistent PSDs representative of nvPM (Section S1 of the Supporting Information).

### GMD and GSD at the Engine Exit Plane

To accurately determine the GMD and GSD at the EEP, the measured PSDs were corrected for size-dependent particle losses in the sampling and measurement systems. This correction involved modeling penetration efficiencies following the methodology described in SAE ARP 6481^29^ and further detailed in previous studies.^21,42^ The internal losses and charging efficiencies accounted for by the sizing instruments were used without any additional corrections.

In this work, we report the log-normal GMD and GSD at the EEP (GMD_EEP_, GSD_EEP_)^10^ to align with log-normal PSD assumptions typically used in emission and local air quality modeling studies.^30,32,33^ Additionally, this assumption reduces the uncertainty that might arise from using different size measurement techniques. As the diluted exhaust plume cools down, volatile PM forms, but the nvPM PSD is conserved.^43^ Therefore, the measured nvPM GMD_EEP_ and GSD_EEP_ are crucial for modeling contrail formation and assessing air quality impacts. These parameters were derived by fitting/minimizing the product of a log-normal distribution and penetration efficiency between the EEP and the instrument against the measured PSD (Section S2 of the Supporting Information).

### System Loss Correction for nvPM Mass and Number

A significant fraction of the nvPM number in the exhaust sample is lost to the inner walls of the sampling and measurement system (total sample line length of up to 35 m), mostly due to diffusion and thermophoresis. The nvPM mass and number concentrations were corrected to the EEP using the regulatory system loss correction method.^9^ This method, which, as discussed previously, does not utilize PSD measurement, requires several assumptions (e.g., particle density, log-normality, GSD) in conjunction with the N/M ratio. Although this method has known uncertainties at low nvPM mass concentration (<10 μg/m^3^ at the instrument) and GMD < ∼20 nm^10^, it was used to be consistent with the nvPM EIs reported by engine manufacturers in the ICAO EEDB. A comparison of the regulatory loss correction factors and correction factors based on measured PSD can be found in Section S3 of the Supporting Information.

### Average nvPM Density Calculation

The average particle effective density was determined by dividing the measured nvPM mass by the volume derived from the PSD measurement at standard temperature and pressure (STP, 0 °C, 101.325 kPa). The volume was calculated by converting the number-weighted PSD into volume-space assuming sphericity and then fitting a log-normal distribution onto the volume-weighted PSD only using measured data <300 nm to prevent DMS500 noise from impacting the results.^10,44^ The total volume was derived by integrating the fitted log-normal distributions <1000 nm (size cutoff for the cyclone in nvPM systems). Additionally, the number concentration reported by the sizing instruments was normalized to the concentration reported by the AVL APC (corrected for additional losses in the APC). Although recent comparisons on an RQL rig have shown excellent agreement (slopes within 5% of the 1:1 line) of the number concentrations reported by the AVL APCs and the sizing instruments in the EUR and CHF systems,^40^ the agreement during the engine tests over the years varied with slopes within 25% of the 1:1 line. Details of the density calculation and comparison with the values obtained with the number concentration reported by the sizing instruments can be found in Section S4 of the Supporting Information.

### Data Averaging and Cleaning

The data were averaged over stable test periods between 30 and 60 s. Data points were excluded when the determined GMD_EEP_ was <7 nm (high measurement and loss correction uncertainty) and when the nvPM mass concentration measured was affected by shedding of large particles re-entrained from the nvPM system cyclone separator^10^ (i.e., the nvPM mass measured included excess nvPM not originating from the engine). In addition to cleanliness checks performed during the tests (system background measurement with pure diluent gas), various metrics were employed in diagnosing cleanliness issues in the data collected based on operational experience. Cleanliness issues during nvPM testing are coindicated by an unreasonably high average nvPM effective density, above the inherent material density of soot of ∼1.8 g/cm^3^,^45^ and GMD_EEP_ predicted by the regulatory systems loss correction method notably larger than the one derived from PSD measurements.^10^ The cleanliness issues are exacerbated by the high measurement uncertainty of the nvPM mass close to the limit of detection (1 μg/m^3^). The measurement uncertainties estimated for all characteristics derived from measured nvPM mass, nvPM number, and PSD are provided in Section S5 of the Supporting Information.

## Results and Discussion

### GMD_EEP_ and GSD_EEP_ as a Function of Engine Thrust

**Figure 1:**
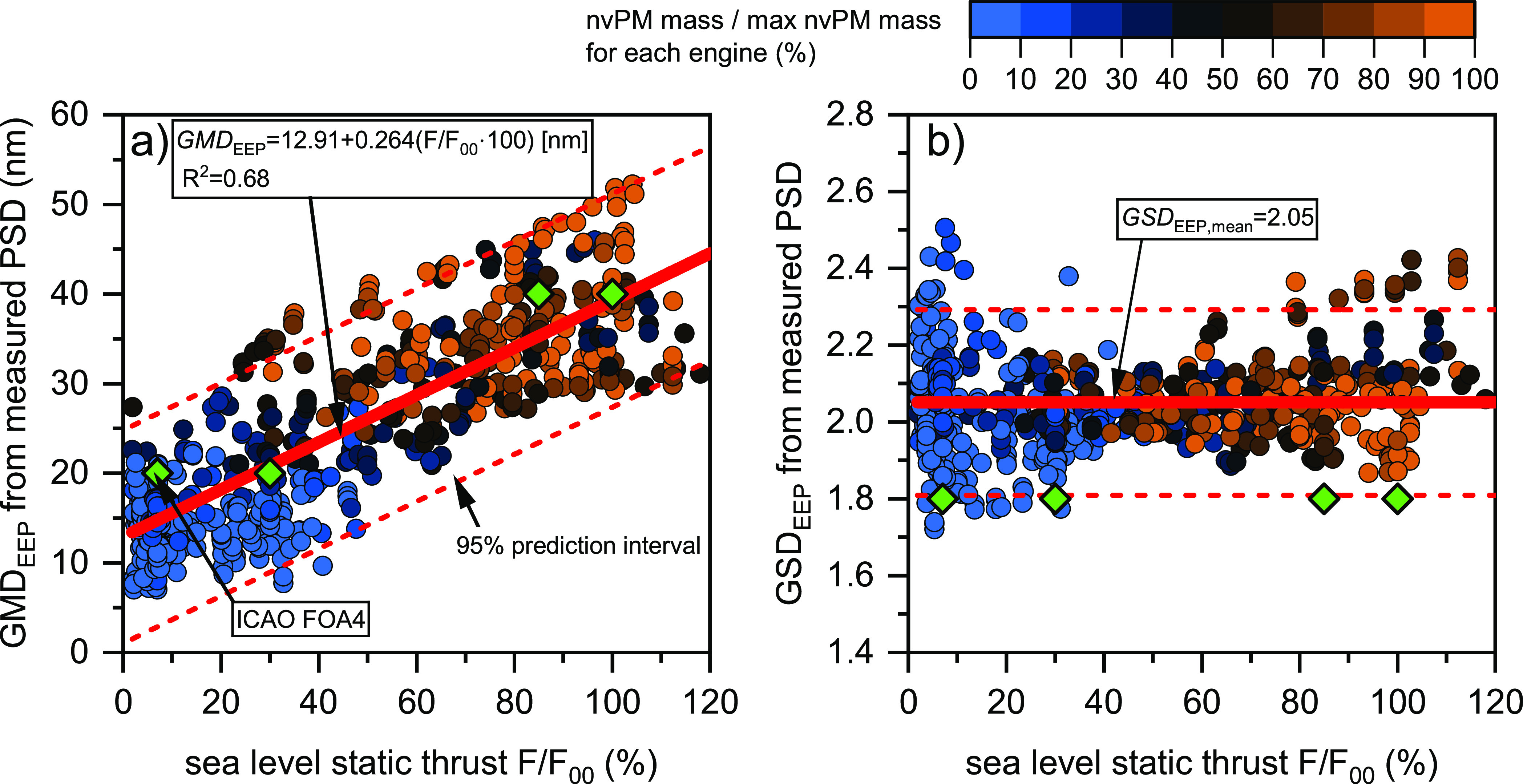
Log-normal engine exit plane nvPM GMD (GMD_EEP_, a) and GSD (GSD_EEP_, b) derived from PSD measurement as a function of engine thrust. Color mapping represents the dilution-corrected normalized nvPM mass concentration for each engine. The green diamonds correspond to the FOA4 assumptions.

The geometric mean diameter at the engine exit plane consistently increased with thrust across all of the tested engines (Figure 1a). However, the GMD_EEP_ values and thrust dependence varied significantly between different engine types (Section S6 of the Supporting Information). For the combined data set, a linear regression without weighting was fitted to allow GMD_EEP_ predictions as a function of engine thrust (eq 1, *R*^2^=0.68):
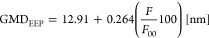
1where *F*/*F*_00_ is the thrust setting relative to the rated takeoff thrust *F*_00_. According to this linear model, the predicted GMD_EEP_ at the regulatory LTO thrust levels is 15 nm at idle (7% thrust), 21 nm at approach (30% thrust), 35 nm at climb (85% thrust), and 39 nm at takeoff (100% thrust). These predicted GMD_EEP_ values are within 5 nm of the assumptions used in the FOA4 method, as outlined in the ICAO Airport Air Quality Manual,^31^ for estimating nvPM number EIs from certified SN data (Figure 1a, green diamonds). The assumed GMD is a major source of uncertainty in the FOA4 method. For instance, using a 15 nm GMD at idle instead of the currently prescribed 20 nm results in an nvPM number EI that is ∼2.5 times higher. Similarly, using 35 nm GMD for climb instead of the FOA4-prescribed 40 nm results in an ∼50% higher nvPM number EI. The demonstrated linear relationship between GMD_EEP_ and thrust not only aids in refining the FOA4 assumptions for the LTO cycle but also has the potential to improve the accuracy of airport emission inventories beyond these four reference points.

Although the tested engines generated the largest GMD_EEP_ at maximum thrust, they exhibited maximum nvPM mass concentrations over a wide range of thrust levels. The color mapping in Figure 1 illustrates that peak nvPM mass concentrations were found at thrust levels between 30% and maximum. This finding contrasts with the parametrization in the SCOPE11 method, which correlates GMD_EEP_ with nvPM mass concentration at the combustor exit.^30^ When an engine generates maximum nvPM mass at a low thrust level, the SCOPE11 method predicts the largest GMD_EEP_ at this condition, which contrasts with our findings in Figure 1. It should be noted that the GMD_EEP_ in SCOPE11 is not based on PSD measurements but derived from measured nvPM mass and number, assuming a log-normal distribution.

In contrast to GMD_EEP_, the GSD_EEP_ of the combined data set did not display consistent thrust dependence (Figure 1b). The GSD_EEP_ increased with thrust for some engine types, similar to previous studies of a limited number of in-service turbofan engine types.^11,16^ However, some engine types demonstrated an opposite trend or more complex thrust dependence that had not been reported previously (Section S6 of the Supporting Information). For the combined data set, the GSD_EEP_ remained nearly constant across all thrust levels and GMD_EEP_, with an average value of 2.05. This figure is higher than the 1.8 assumed in FOA4 and the SAE ARP6481 loss correction methodology.

The observed range of GMD_EEP_ and its dependence on thrust are consistent with previous studies that report GMD_EEP_ derived from PSD measurements using DMS500 and SMPS behind large turbofan engines with rich-burn combustors.^8,11,16,18^ However, the linear scaling of GMD_EEP_ with thrust may not be applicable for engines with staged or premixed lean-burn combustion systems (double annular combustor (DAC) and twin annular premixed swirler (TAPS) technology from General Electric).^38^ Given the lack of published data on the emission characteristics of these systems on the ground and in flight, further research is required to determine appropriate predictive correlations, especially considering their increasing numbers in service.

### GMD_EEP_, GSD_EEP_, and Average nvPM Density as a Function of (N/M)_SL_

**Figure 2:**
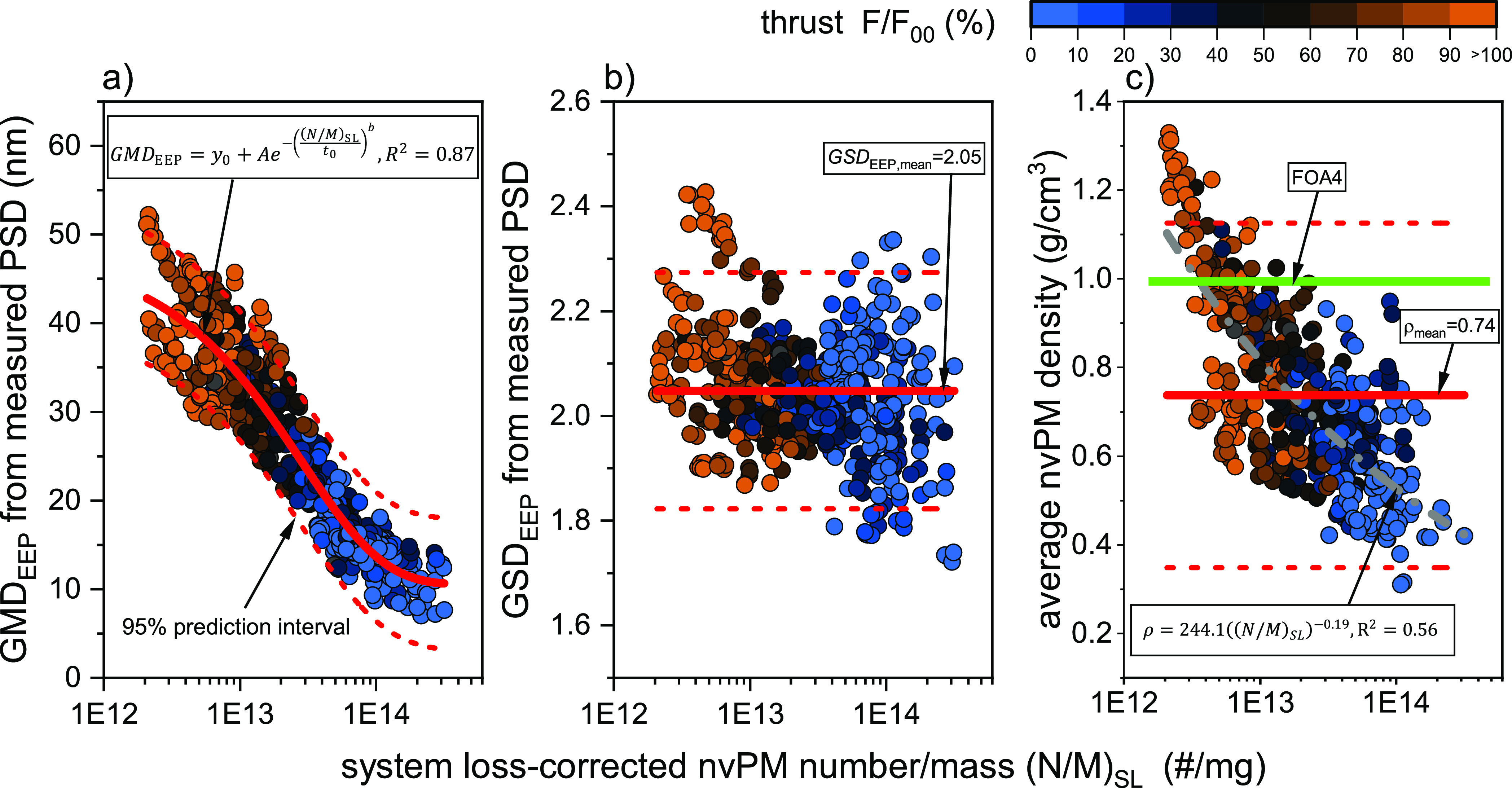
Engine exit plane nvPM GMD (GMD_EEP_, a), GSD (GSD_EEP_, b), and average nvPM density (c) as a function of the ratio of regulatory system loss (SL) corrected nvPM number to mass (N/M). The average density is plotted only for nvPM mass concentrations >5 μg/m^3^ at the instrument due to high measurement uncertainties at low mass concentrations.

Figure 2 examines the relationships of GMD_EEP_, GSD_EEP_, and average nvPM density with the ratio of nvPM number to mass, corrected for sampling system losses using the regulatory loss correction method, (N/M)_SL_. (N/M)_SL_ was selected as it is available in the ICAO EEDB and facilitates GMD_EEP_ derivation under the assumption of log-normal distribution with a fixed GSD_EEP_ and average particle density (see eq 2).

The GMD_EEP_ negatively correlated with (N/M)_SL_ (Figure 2a), with the data best fitted using a stretched exponential function with GMD_EEP_ bounds between 10.5 and 48 nm and an *R*^2^ value of 0.87 (detailed fit parameters are provided in Table 1), even though GMD_EEP_ of individual engines ranged from 7 to 52 nm. This strong GMD_EEP_ correlation with (N/M)_SL_ is applicable across rich-burn engines included in this study despite their varied nvPM emission characteristics. Additionally, the color map in Figure 2a demonstrates a thrust dependency of (N/M)_SL_, with a decrease in (N/M)_SL_ corresponding with increases in thrust and GMD_EEP_. The variance observed around the trendline can be attributed to differences in particle morphology due to engine technology, measurement uncertainties (encompassing nvPM mass, number, and PSD), and uncertainties in the regulatory system loss correction method.

**Table 1:** Parameters Used in the Predictive Models of GMD_EEP_

**model**	**parameter**	**value**
log-normal model 1	GSD	1.8
	ρ	1.0
log-normal model 2	GSD	2.05
	ρ	0.74
exponential fit	*y*_0_	10.52
	*A*	37.5
	*t*_0_	2.86e13
	*b*	0.724

The lower cutoff selection for the nvPM number measurement and standardized loss correction method could affect the GMD_EEP_–(N/M)_SL_ relationship reported here. The 10 nm cutoff was selected in the regulatory nvPM measurement and loss correction methods because of the high measurement uncertainties of sub-10 nm particles with penetration efficiencies <10%.^10^ Should future measurement systems achieve lower uncertainties, it would be feasible to decrease the cutoff. Applying a novel loss correction method with PSD measurement^10^ to our data set revealed that a 7 nm cutoff increases the (N/M)_SL_. As expected, the effect is strongest at the smallest GMD_EEP_. For example, at a GMD_EEP_ of 10 nm, the (N/M)_SL_ is ∼20% higher. However, this adjustment to the lower cutoff would have a negligible effect (<1 nm) on the predicted GMD_EEP_ as a function of (N/M)_SL._

In contrast, GSD_EEP_, ranging from 1.7 to 2.5, showed no substantial trend with (N/M)_SL_ or GMD_EEP_ (Figure 2b). A linear regression suggests a minor decrease in GSD_EEP_ with (N/M)_SL_ from 2.1 to 2.00 within the studied (N/M)_SL_ range.

The average nvPM density varied considerably, ranging from 0.30 to 1.35 g/cm^3^, and had a pronounced dependency on (N/M)_SL_ and thrust (Figure 2c). Contrary to the assumption of unit particle effective density (1 g/cm^3^) in FOA4 and the regulatory system loss correction methodology, the average nvPM density for the combined data set was 0.74 g/cm^3^. Higher densities were typically observed at lower (N/M)_SL_, which correspond to high thrust conditions, with variability thought to be influenced mainly by varying the soot morphology and primary particle size across different engine types and operating conditions. Notably, the size instruments did not exhibit bias in GMD_EEP_, GSD_EEP_, and calculated densities (Section S4 of the Supporting Information).

The decreasing average nvPM density with increasing (N/M)_SL_ (i.e., decreasing thrust) contrasts with previous studies that calculated the average particle effective density using integrated PSD and size-dependent effective densities. Previous investigations of a large turbofan engine and a small turbojet reported average densities between 0.6 and 1.0 g/cm^3^, slightly decreasing with increasing thrust.^46,47^ The differences may lay in the inherently different definition of the average nvPM density used here (including the log-normality assumption). The average nvPM density used here depends on the optical properties of soot, which vary with the thrust. The nvPM mass in this study was measured as equivalent black carbon (eBC) calibrated to the elemental carbon (EC) content of soot, in line with regulations.^48^ The EC fraction of aircraft engine soot has been found to increase with thrust, with a maximum value of ∼90%,^49^ which was also confirmed visually where particles collected at idle were brown whereas high-thrust particles were black.^50^ Consequently, the optical nvPM mass measurement may significantly underreport the total PM mass obtained from integrated PSD and size-dependent density distributions, as reported by Giannelli et al.^47^ Nevertheless, the methodology for determining the average nvPM density adopted in this study, along with the reported values, is appropriate in the context of regulatory nvPM measurements and the relationship between the regulatory nvPM mass and number.

### Comparison of GMD_EEP_ Predicted and Derived from PSD Measurement

**Figure 3:**
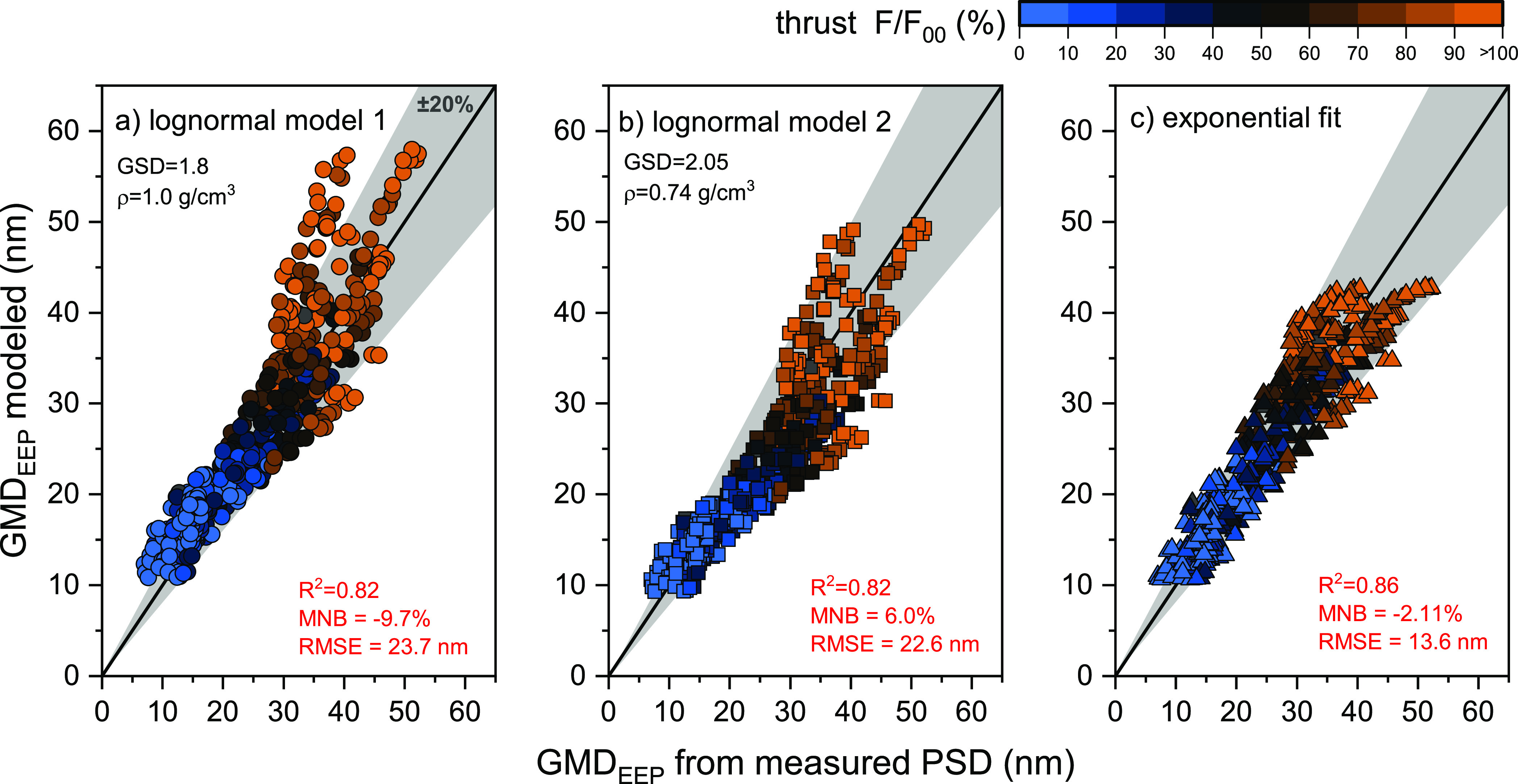
Parity plots between GMD_EEP_ predicted and derived from PSD measurement. Log-normal model 1 (a) assumes GSD = 1.8 and an average density of 1 g/cm^3^. Log-normal model 2 (b) utilizes the average GSD and density from Figure 2. The stretched exponential fit (c) represents the best fit of the data in this study.

Figure 3 compares the GMD_EEP_ derived from PSD measurements with that predicted by log-normal models from the literature^30^ (eq 2) and the exponential fit proposed in Figure 2a (eq 3). The log-normal models utilize (N/M)_SL_ available in the ICAO EEDB, along with an assumed GSD and average particle effective density as input parameters.
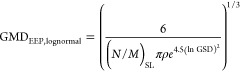
2

As listed in Table 1, log-normal model 1 (Figure 3a) uses standard regulatory values for GSD and density, whereas log-normal model 2 (Figure 3b) incorporates the mean GSD_EEP_ and density derived from the PSD measurements (Figure 2a,b).

The stretched exponential function (best fit to the empirical data) shown in Figure 2a is parametrized as
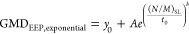
3

The fit parameters for this function are also listed in Table 1.

Both log-normal models demonstrate a good correlation with the empirical data with an *R*^2^=0.82 and accurately approximate GMD_EEP_ within the 20 to 40 nm range. This range is consistent with in-flight measurements behind a commercial turbofan engine burning fossil jet fuel and biofuel blends.^20^ However, uncertainties increase for GMD_EEP_ outside this range, with many data points falling beyond the ±20% bands. Attempts to refine the model, such as incorporating a density function dependent on (N/M)_SL_ (as shown in Figure 2c), adjusted the parity plot slope but did not reduce the data scatter. This scatter is mainly influenced by the uncertainties in the reported nvPM number and mass used in the GMD model and the variations in the particle morphology across different engine types and thrust settings.

The stretched exponential fit derived from PSD measurements offers the best overall agreement with our empirical data. As shown in Figure 3, this model has the highest *R*^2^ (0.86) and the lowest mean normalized bias (MNB, −2.11%) and root-mean-square error (RMSE, 13.6 nm). However, it is important to note that this fit is constrained by the range of experimental data and engines used, limiting predicted GMD_EEP_ to a range of 10.5–48 nm, with a tendency to underpredict measured GMD_EEP_ > 40 nm. This range is representative of GMD_EEP_ typically produced by most commercial turbofan engines across all thrust settings burning conventional Jet A or Jet A-1 fuel.

### Application of the GMD_EEP_ Models to the (N/M)_SL_ Data in the ICAO EEDB

**Figure 4:**
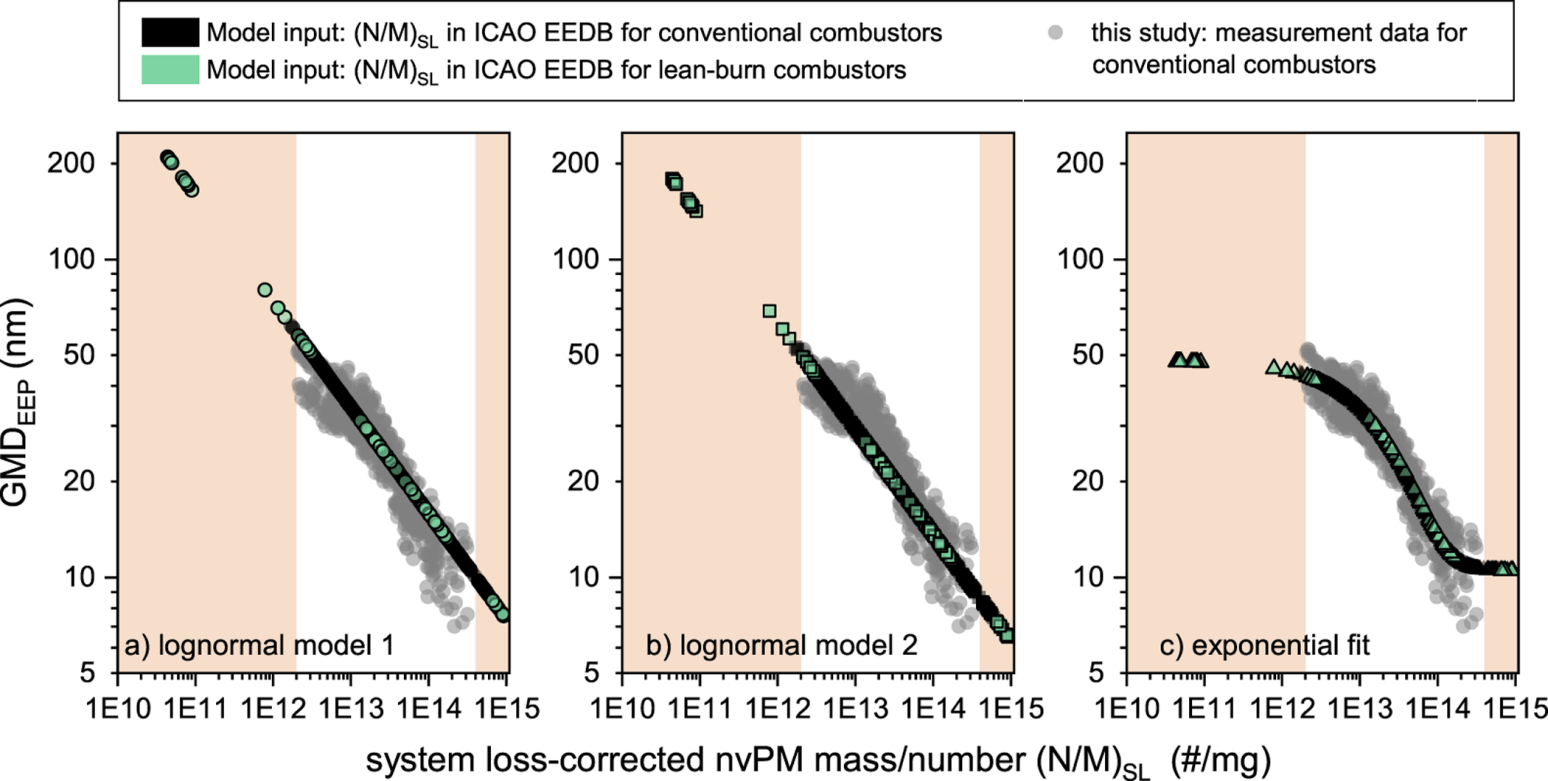
nvPM GMD_EEP_ predicted using log-normal model 1 (a), log-normal model 2 (b), and the exponential fit to the empirical data (c) as a function of regulatory system loss (SL) corrected N/M taken from the ICAO Engine Emissions Databank. The shaded areas indicate extrapolations beyond empirical data in this study.

The three models were applied to system loss-corrected N/M data from the ICAO Engine Emissions Databank, version 29b, to predict GMD_EEP_ of certified engines (Figure 4).^27^ The (N/M)_SL_ was directly derived from the nvPM number and mass EIs reported in the EEDB corrected for system loss using the regulatory method with a 10 nm cutoff.^29^ The models were applied to EEDB data for both conventional rich-burn and lean-burn combustor engines. However, it is important to note that our analysis with lean-burn engines primarily highlights potential limitations, as no experimental data were collected for these engines.

The empirical data in this study spanned (N/M)_SL_ values from 2e12 to 3e14 particles/mg, covering most of the range for conventional combustors in the EEDB (black symbols in Figure 4), with the shaded areas in Figure 4 highlighting the range not covered experimentally. The highest (N/M)_SL_ for conventional combustors in the EEDB was ∼9e14 particles/mg at 7% thrust of Pratt & Whitney engines equipped with the TALON X combustor.^51^ These entries, with a high nvPM number but low nvPM mass EIs, had the smallest predicted GMD_EEP_. Log-normal model 2 estimated GMD_EEP_ as small as 6.5 nm, whereas the exponential fit estimated a more conservative lower limit of 10.5 nm. The lowest (N/M)_SL_ reported was ∼2e12 at the takeoff thrust of a Rolls-Royce Trent 1000 engine, for which the log-normal model 1 predicted GMD_EEP_ of ∼60 nm. This value is larger than any GMD_EEP_ found in this study and it is likely an overestimation (Figure 3a). The exponential fit predicted a GMD_EEP_ of ∼43 nm, in line with the empirical data.

For lean-burn engines with DAC and TAPS combustors, the (N/M)_SL_ values and GMD_EEP_ predictions at 7% thrust were similar to conventional combustors since at low thrust, lean-burn engines operate with a rich-burn primary (pilot) zone.^38^ The highest (N/M)_SL_ was also ∼9e14 with a predicted GMD_EEP_ as small as 6.5 nm (CFM LEAP-1B engine). However, at high thrust (85% and 100% thrust), the reported (N/M)_SL_ for engines featuring the TAPS combustor decreases dramatically <3e12, up to 3 orders of magnitude, as shown by the green symbols in the left shaded area in Figure 4. The low (N/M)_SL_ for lean-burn engines at high thrust is driven by the nvPM mass concentrations being at ambient levels and below the limit of quantification of regulatory nvPM systems, which poses challenges for accurate modeling. For the lowest reported (N/M)_SL_ values for these engines, ∼ 2e10 particles/mg, the extrapolated log-normal models predicted implausibly large GMD_EEP_ of up to ∼200 nm (GEnx and CFM LEAP-1A engines), which, to the authors’ knowledge, has never been witnessed in a gas turbine exhaust across any engine technology. The exponential fit predicted a GMD_EEP_ of ∼47 nm, which is also likely an overestimation.

Previous studies of a DAC engine (TAPS predecessor) suggest that lean-burn engines might exhibit a different GMD_EEP_ versus (N/M)_SL_ relationship across various operating regimes^13,52^: At the ground, the GMD_EEP_ for these engines increases steeply from ∼10 to ∼30 nm when the thrust increases from idle to ∼30%. In this thrust range, only the rich-burn primary zone is active. When the thrust is increased further, the main lean-burn zone is activated, and nvPM mass and number drop by several orders of magnitude. The GMD_EEP_ drops to ∼15 nm and remains constant with a further increase in the thrust.

Overall, the exponential model (Figure 4c) improves the prediction of PSD characteristics from preexisting ICAO certification data sets for conventional engines. Compared to the log-normal models, it also restricts the GMD_EEP_ to a realistic range aligned with experimental studies of gas turbine engine emissions. The applicability of this model can be further extended and validated to novel engine technologies and fuels when nvPM emissions data complemented by PSD measurements become available.

## Supporting Information Available

PSD measurements with and without a catalytic stripper (Section S1); determination of the GMD and GSD at the engine exit plane (Section S2); comparison of system loss correction factors with and without PSD measurement (Section S3); average nvPM density calculation (Section S4); measurement uncertainty estimation (Section S5); : nvPM GMD_EEP_ and GSD_EEP_ as a function thrust and engine type (Section S6) (PDF)
Tables with data (XLSX)

## References

[ref1] Lee H.; Olsen S. C.; Wuebbles D. J.; Youn D. Impacts of Aircraft Emissions on the Air Quality near the Ground. Atmos. Chem. Phys. 2013, 13 (11), 5505–5522. 10.5194/acp-13-5505-2013.

[ref2] Kärcher B. Formation and Radiative Forcing of Contrail Cirrus. Nat. Commun. 2018, 9 (1), 1824 10.1038/s41467-018-04068-0. 29739923 PMC5940853

[ref3] Bendtsen K. M.; Bengtsen E.; Saber A. T.; Vogel U. A Review of Health Effects Associated with Exposure to Jet Engine Emissions in and around Airports. Environ. Health 2021, 20 (1), 10 10.1186/s12940-020-00690-y. 33549096 PMC7866671

[ref4] Lee D. S.; Fahey D. W.; Skowron A.; Allen M. R.; Burkhardt U.; Chen Q.; Doherty S. J.; Freeman S.; Forster P. M.; Fuglestvedt J.; Gettelman A.; De León R. R.; Lim L. L.; Lund M. T.; Millar R. J.; Owen B.; Penner J. E.; Pitari G.; Prather M. J.; Sausen R.; Wilcox L. J. The Contribution of Global Aviation to Anthropogenic Climate Forcing for 2000 to 2018. Atmos. Environ. 2021, 244, 117834 10.1016/j.atmosenv.2020.117834. PMC7468346 32895604

[ref5] Lee D. S.; Allen M. R.; Cumpsty N.; Owen B.; Shine K. P.; Skowron A. Uncertainties in Mitigating Aviation Non-CO_2_ Emissions for Climate and Air Quality Using Hydrocarbon Fuels. Environ. Sci.: Atmos. 2023, 3 (12), 1693–1740. 10.1039/D3EA00091E.

[ref6] Hagen D. E.; Trueblood M. B.; Whitefield P. D. A Field Sampling of Jet Exhaust Aerosols. Particulate Sci. & Technol. 1992, 10 (1), 53–63. 10.1080/02726359208906598.

[ref7] Hagen D.; Whitefield P.; Paladino J.; Trueblood M.; Lilenfeld H. Particulate Sizing and Emission Indices for a Jet Engine Exhaust Sampled at Cruise. Geophys. Res. Lett. 1998, 25 (10), 1681–1684. 10.1029/97GL03504.

[ref8] Lobo P.; Hagen D. E.; Whitefield P. D.; Alofs D. J. Physical Characterization or Aerosol Emissions from a Commercial Gas Turbine Engine. J. Propuls. Power 2007, 23 (5), 919–929. 10.2514/1.26772.

[ref9] ICAO. Annex 16 to the Convention on International Civil Aviation: Environmental Protection, Vol. II – Aircraft Engine Emissions, 4th ed.; ICAO, 2017.

[ref10] Durand E.; Durdina L.; Smallwood G.; Johnson M.; Spirig C.; Edebeli J.; Roth M.; Brem B.; Sevcenco Y.; Crayford A. Correction for Particle Loss in a Regulatory Aviation nvPM Emissions System Using Measured Particle Size. J. Aerosol Sci. 2023, 169, 106140 10.1016/j.jaerosci.2023.106140.

[ref11] Lobo P.; Hagen D. E.; Whitefield P. D.; Raper D. PM Emissions Measurements of In-Service Commercial Aircraft Engines during the Delta-Atlanta Hartsfield Study. Atmos. Environ. 2015, 104, 237–245. 10.1016/j.atmosenv.2015.01.020.

[ref12] Lobo P.; Christie S.; Khandelwal B.; Blakey S. G.; Raper D. W. Evaluation of Non-Volatile PM Emissions Characteristics of an Aircraft Auxiliary Power Unit with Varying Alternative Jet Fuel Blend Ratios. Energy Fuels 2015, 29, 7705 10.1021/acs.energyfuels.5b01758.

[ref13] Lobo P.; Durdina L.; Smallwood G. J.; Rindlisbacher T.; Siegerist F.; Black E. A.; Yu Z.; Mensah A. A.; Hagen D. E.; Miake-Lye R. C.; Thomson K. A.; Brem B. T.; Corbin J. C.; Abegglen M.; Sierau B.; Whitefield P. D.; Wang J. Measurement of Aircraft Engine Non-Volatile PM Emissions: Results of the Aviation-Particle Regulatory Instrumentation Demonstration Experiment (A-PRIDE) 4 Campaign. Aerosol Sci. Technol. 2015, 49 (7), 472–484. 10.1080/02786826.2015.1047012.

[ref14] Lobo P.; Durdina L.; Brem B. T.; Crayford A. P.; Johnson M. P.; Smallwood G. J.; Siegerist F.; Williams P. I.; Black E. A.; Llamedo A.; Thomson K. A.; Trueblood M. B.; Yu Z.; Hagen D. E.; Whitefield P. D.; Miake-Lye R. C.; Rindlisbacher T. Comparison of Standardized Sampling and Measurement Reference Systems for Aircraft Engine Non-Volatile Particulate Matter Emissions. J. Aerosol Sci. 2020, 145, 105557 10.1016/j.jaerosci.2020.105557.

[ref15] Kinsey J. S.; Dong Y.; Williams D. C.; Logan R. Physical Characterization of the Fine Particle Emissions from Commercial Aircraft Engines during the Aircraft Particle Emissions eXperiment (APEX) 1–3. Atmos. Environ. 2010, 44 (17), 2147–2156. 10.1016/j.atmosenv.2010.02.010. 21428391

[ref16] Corbin J. C.; Schripp T.; Anderson B. E.; Smallwood G. J.; LeClercq P.; Crosbie E. C.; Achterberg S.; Whitefield P. D.; Miake-Lye R. C.; Yu Z.; Freedman A.; Trueblood M.; Satterfield D.; Liu W.; Oßwald P.; Robinson C.; Shook M. A.; Moore R. H.; Lobo P. Aircraft-Engine Particulate Matter Emissions from Conventional and Sustainable Aviation Fuel Combustion: Comparison of Measurement Techniques for Mass, Number, and Size. Atmos. Meas. Technol. 2022, 15 (10), 3223–3242. 10.5194/amt-15-3223-2022.

[ref17] Durand E.; Lobo P.; Crayford A.; Sevcenco Y.; Christie S. Impact of Fuel Hydrogen Content on Non-Volatile Particulate Matter Emitted from an Aircraft Auxiliary Power Unit Measured with Standardised Reference Systems. Fuel 2021, 287, 119637 10.1016/j.fuel.2020.119637.

[ref18] Durdina L.; Brem B. T.; Elser M.; Schönenberger D.; Siegerist F.; Anet J. G. Reduction of Nonvolatile Particulate Matter Emissions of a Commercial Turbofan Engine at the Ground Level from the Use of a Sustainable Aviation Fuel Blend. Environ. Sci. Technol. 2021, 55 (21), 14576–14585. 10.1021/acs.est.1c04744. 34662519

[ref19] Durdina L.; Brem B. T.; Schönenberger D.; Siegerist F.; Anet J. G.; Rindlisbacher T. Non-Volatile Particulate Matter Emissions of a Business Jet Measured at Ground Level and Estimated for Cruising Altitudes. Environ. Sci. Technol. 2019; DOI 53 12865 10.1021/acs.est.9b02513. 31578862

[ref20] Moore R. H.; Thornhill K. L.; Weinzierl B.; Sauer D.; D’Ascoli E.; Kim J.; Lichtenstern M.; Scheibe M.; Beaton B.; Beyersdorf A. J.; Barrick J.; Bulzan D.; Corr C. A.; Crosbie E.; Jurkat T.; Martin R.; Riddick D.; Shook M.; Slover G.; Voigt C.; White R.; Winstead E.; Yasky R.; Ziemba L. D.; Brown A.; Schlager H.; Anderson B. E. Biofuel Blending Reduces Particle Emissions from Aircraft Engines at Cruise Conditions. Nature 2017, 543 (7645), 411–415. 10.1038/nature21420. 28300096 PMC8025803

[ref21] Kittelson D. B.; Swanson J.; Aldridge M.; Giannelli R. A.; Kinsey J. S.; Stevens J. A.; Liscinsky D. S.; Hagen D.; Leggett C.; Stephens K.; Hoffman B.; Howard R.; Frazee R. W.; Silvis W.; McArthur T.; Lobo P.; Achterberg S.; Trueblood M.; Thomson K.; Wolff L.; Cerully K.; Onasch T.; Miake-Lye R.; Freedman A.; Bachalo W.; Payne G. Experimental Verification of Principal Losses in a Regulatory Particulate Matter Emissions Sampling System for Aircraft Turbine Engines. Aerosol Sci. Technol. 2022, 56 (1), 63–74. 10.1080/02786826.2021.1971152. PMC9118390 35602286

[ref22] Delhaye D.; Ouf F.-X.; Ferry D.; Ortega I. K.; Penanhoat O.; Peillon S.; Salm F.; Vancassel X.; Focsa C.; Irimiea C.; Harivel N.; Perez B.; Quinton E.; Yon J.; Gaffie D. The MERMOSE Project: Characterization of Particulate Matter Emissions of a Commercial Aircraft Engine. J. Aerosol Sci. 2017, 105, 48–63. 10.1016/j.jaerosci.2016.11.018.

[ref23] Schripp T.; Anderson B. E.; Bauder U.; Rauch B.; Corbin J. C.; Smallwood G. J.; Lobo P.; Crosbie E. C.; Shook M. A.; Miake-Lye R. C.; Yu Z.; Freedman A.; Whitefield P. D.; Robinson C. E.; Achterberg S. L.; Köhler M.; Oßwald P.; Grein T.; Sauer D.; Voigt C.; Schlager H.; LeClercq P. Aircraft Engine Particulate Matter Emissions from Sustainable Aviation Fuels: Results from Ground-Based Measurements during the NASA/DLR Campaign ECLIF2/ND-MAX. Fuel 2022, 325, 124764 10.1016/j.fuel.2022.124764.

[ref24] EASA. Introduction to the ICAO Engine Emissions Databank; EASA: 2023; https://www.easa.europa.eu/en/downloads/45576/en (accessed May 13, 2024).

[ref25] Crayford A.; Johnson M.; Sevcenco Y.; Williams P. SAMPLE III SC.05 - Studying, sAmpling and Measuring of Aircraft ParticuLate Emission. 2014. https://www.easa.europa.eu/document-library/research-reports/easa2010fc10-sc05 (accessed May 13, 2024).

[ref26] Lister D. H.; Norman P. D. EC-NEPAir: Work Package 1 Aircraft Engine Emissions Certification - a Review of the Development of ICAO Annex 16, *Volume II*; QINETIQ/FST/CR030440; 2003; p 236.

[ref27] EASA. ICAO Aircraft Engine Emissions Databank v29b; EASA: 2023. https://www.easa.europa.eu/en/domains/environment/icao-aircraft-engine-emissions-databank (accessed May 13, 2024).

[ref28] SAE International. Aerospace Information Report (AIR) 6504 - Procedure for the Calculation of Sampling System Penetration Functions and System Loss Correction Factors; SAE International, 2022 10.4271/AIR6504.

[ref29] SAE International. Aerospace Recommended Practice (ARP) 6481 - Procedure for the Calculation of Sampling Line Penetration Functions and Line Loss Correction Factors; SAE International, 2019 10.4271/ARP6481.

[ref30] Agarwal A.; Speth R. L.; Fritz T. M.; Jacob S. D.; Rindlisbacher T.; Iovinelli R.; Owen B.; Miake-Lye R. C.; Sabnis J. S.; Barrett S. R. H. SCOPE11 Method for Estimating Aircraft Black Carbon Mass and Particle Number Emissions. Environ. Sci. Technol. 2019, 53 (3), 1364–1373. 10.1021/acs.est.8b04060. 30620574

[ref31] ICAO. Doc 9889, Airport Air Quality Manual, 2nd ed.; ICAO, 2020. https://www.icao.int/publications/documents/9889_cons_en.pdf (accessed May 13, 2024).

[ref32] Teoh R.; Stettler M. E. J.; Majumdar A.; Schumann U.; Graves B.; Boies A. M. A Methodology to Relate Black Carbon Particle Number and Mass Emissions. J. Aerosol Sci. 2019, 132, 44–59. 10.1016/j.jaerosci.2019.03.006.

[ref33] Ahrens D.; Méry Y.; Guénard A.; Miake-Lye R. C. A New Approach to Estimate Particulate Matter Emissions From Ground Certification Data: The nvPM Mission Emissions Estimation Methodology. J. Eng. Gas Turbines and Power 2023, 145 (3), 031019 10.1115/1.4055477.

[ref34] Teoh R.; Schumann U.; Voigt C.; Schripp T.; Shapiro M.; Engberg Z.; Molloy J.; Koudis G.; Stettler M. E. J. Targeted Use of Sustainable Aviation Fuel to Maximize Climate Benefits. Environ. Sci. Technol. 2022, 56 (23), 17246–17255. 10.1021/acs.est.2c05781. 36394538 PMC9730838

[ref35] Voigt C.; Kleine J.; Sauer D.; Moore R. H.; Bräuer T.; Le Clercq P.; Kaufmann S.; Scheibe M.; Jurkat-Witschas T.; Aigner M.; Bauder U.; Boose Y.; Borrmann S.; Crosbie E.; Diskin G. S.; DiGangi J.; Hahn V.; Heckl C.; Huber F.; Nowak J. B.; Rapp M.; Rauch B.; Robinson C.; Schripp T.; Shook M.; Winstead E.; Ziemba L.; Schlager H.; Anderson B. E. Cleaner Burning Aviation Fuels Can Reduce Contrail Cloudiness. Commun. Earth. Environ. 2021, 2 (1), 114 10.1038/s43247-021-00174-y.

[ref36] Kärcher B.; Burkhardt U.; Bier A.; Bock L.; Ford I. J. The Microphysical Pathway to Contrail Formation. JGR Atmospheres 2015, 120 (15), 7893–7927. 10.1002/2015JD023491.

[ref37] Jones S. H.; Miake-Lye R. C. Contrail Modeling of ECLIF2/ND-MAX Flights: Effects of nvPM Particle Numbers and Fuel Sulfur Content. metz 2023, 103024 10.1127/metz/2023/1180.

[ref38] Stickles R.; Barrett J. TAPS II Combustor Final Report; DTFAWA-10-C-00046; 2013. https://www.faa.gov/sites/faa.gov/files/about/office_org/headquarters_offices/apl/TAPS_II_Public_Final_Report.pdf (accessed May 13, 2024).

[ref39] SAE International. Aerospace Recommended Practice (ARP) 6320A - Procedure for the Continuous Sampling and Measurement of Non-Volatile Particulate Matter Emissions from Aircraft Turbine Engines; SAE International, 2021 10.4271/ARP6320.

[ref40] Crayford A.; Durand E.; Delhaye D.; Durdina L.; Ortega I. K.; Williams P. RAPTOR Work Package 4: PM Measurements Deliverables Report; 2022. https://zenodo.org/records/7385796 (accessed May 13, 2024).

[ref41] TSI. Application Note SMPS-006: Fast Scanning Using TSI’s Scanning Mobility Particle SizerTM (SMPSTM) Spectrometer Model 3938; TSI, 2013. https://tsi.com/getmedia/a28ed8a9-474b-45ad-9bf9-ab08750673fa/SMPS-006_Fast_Scanning_Using_3938-web?ext=.pdf (accessed May 13, 2024).

[ref42] Durand E. F.; Crayford A. P.; Johnson M. Experimental Validation of Thermophoretic and Bend Nanoparticle Loss for a Regulatory Prescribed Aircraft nvPM Sampling System. Aerosol Sci. Technol. 2020, 54 (9), 1019–1033. 10.1080/02786826.2020.1756212.

[ref43] Beyersdorf A. J.; Timko M. T.; Ziemba L. D.; Bulzan D.; Corporan E.; Herndon S. C.; Howard R.; Miake-Lye R.; Thornhill K. L.; Winstead E.; Wey C.; Yu Z.; Anderson B. E. Reductions in Aircraft Particulate Emissions Due to the Use of Fischer–Tropsch Fuels. Atmos. Chem. Phys. 2014, 14 (1), 11–23. 10.5194/acp-14-11-2014.

[ref44] Cambustion. Particulate Mass Measurement with DMS Series Fast Spectrometers - Cambustion Application Note DMS01v6*;* https://www.cambustion.com/files/1606395876-dms01.pdf (accessed 13 May 2024).

[ref45] Park K.; Kittelson D. B.; Zachariah M. R.; McMurry P. H. Measurement of Inherent Material Density of Nanoparticle Agglomerates. J. Nanoparticle Res. 2004, 6 (2/3), 267–272. 10.1023/B:NANO.0000034657.71309.e6.

[ref46] Durdina L.; Brem B. T.; Abegglen M.; Lobo P.; Rindlisbacher T.; Thomson K. A.; Smallwood G. J.; Hagen D. E.; Sierau B.; Wang J. Determination of PM Mass Emissions from an Aircraft Turbine Engine Using Particle Effective Density. Atmos. Environ. 2014, 99, 500–507. 10.1016/j.atmosenv.2014.10.018.

[ref47] Giannelli R.; Stevens J.; Kinsey J. S.; Kittelson D.; Zelenyuk A.; Howard R.; Forde M.; Hoffman B.; Leggett C.; Maeroff B.; Bies N.; Swanson J.; Suski K.; Payne G.; Manin J.; Frazee R.; Onasch T. B.; Freedman A.; Khalek I.; Badshah H.; Preece D.; Premnath V.; Agnew S. Evaluation of Methods for Characterizing the Fine Particulate Matter Emissions from Aircraft and Other Diffusion Flame Combustion Aerosol Sources. J. Aerosol Sci. 2024, 178, 106352 10.1016/j.jaerosci.2024.106352. PMC11095129 38751612

[ref48] Durdina L.; Lobo P.; Trueblood M. B.; Black E. A.; Achterberg S.; Hagen D. E.; Brem B. T.; Wang J. Response of Real-Time Black Carbon Mass Instruments to Mini-CAST Soot. Aerosol Sci. Technol. 2016, 50 (9), 906–918. 10.1080/02786826.2016.1204423.

[ref49] Elser M.; Brem B. T.; Durdina L.; Schönenberger D.; Siegerist F.; Fischer A.; Wang J. Chemical Composition and Radiative Properties of Nascent Particulate Matter Emitted by an Aircraft Turbofan Burning Conventional and Alternative Fuels. Atmos. Chem. Phys. 2019, 19 (10), 6809–6820. 10.5194/acp-19-6809-2019.

[ref50] Heeb N. V.; Muñoz M.; Haag R.; Wyss S.; Schönenberger D.; Durdina L.; Elser M.; Siegerist F.; Mohn J.; Brem B. T. Corelease of Genotoxic Polycyclic Aromatic Hydrocarbons and Nanoparticles from a Commercial Aircraft Jet Engine – Dependence on Fuel and Thrust. Environ. Sci. Technol. 2024, 58 (3), 1615–1624. 10.1021/acs.est.3c08152. 38206005 PMC10809754

[ref51] McKinney R.; Cheung A.; Sowa W.; Sepulveda D. The Pratt & Whitney TALON X Low Emissions Combustor: Revolutionary Results with Evolutionary Technology. In 45th AIAA Aerospace Sciences Meeting and Exhibit; American Institute of Aeronautics and Astronautics: Reno, NV, 2007 10.2514/6.2007-386.

[ref52] Boies A. M.; Stettler M. E. J.; Swanson J. J.; Johnson T. J.; Olfert J. S.; Johnson M.; Eggersdorfer M. L.; Rindlisbacher T.; Wang J.; Thomson K.; Smallwood G.; Sevcenco Y.; Walters D.; Williams P. I.; Corbin J.; Mensah A. A.; Symonds J.; Dastanpour R.; Rogak S. N. Particle Emission Characteristics of a Gas Turbine with a Double Annular Combustor. Aerosol Sci. Technol. 2015, 49 (9), 842–855. 10.1080/02786826.2015.1078452.

