# Supplementary material for: Characterizing and Predicting nvPM Size Distributions for Aviation Emission Inventories and Environmental Impact
Source: Environ Sci Technol. 2024 Jun 10;58(24):10548–57. doi: 10.1021/acs.est.4c02538 (PMC11191599; doi:10.1021/acs.est.4c02538)
Supplement: Supplementary file 1 — es4c02538_si_001.pdf [file es4c02538_si_001.pdf]

Supporting Information

for

Characterizing and Predicting nvPM Size Distributions for Aviation Emission Inventories and Environmental Impact

Lukas Durdina[*a], Eliot Durand[*b], Jacinta Edebeli[a], Curdin Spirig[a], Benjamin T. Brem[c], Miriam Elser[d], Frithjof Siegerist[e], Mark Johnson[f], Yura A. Sevcenco[b] , Andrew P. Crayford[b]

[a] *Centre for Aviation, School of Engineering, Zurich University of Applied Sciences, Winterthur, Switzerland*
[b] *Cardiff School of Engineering, Cardiff University, Wales, UK*

[c] *Laboratory of Atmospheric Chemistry, Paul Scherrer Institute, Villigen, Switzerland*

[d] *Laboratory for Automotive Powertrain Technologies, Empa, Dübendorf, Switzerland*

[e] *SR Technics Switzerland AG, Zurich-Airport, Switzerland*

[f] *Rolls-Royce,Plc, Derby, UK*

Number of pages: 15
Number of figures: 9
Number of tables: 0

Contents

S1 PSD measurements with and without a catalytic stripper

S2 Determination of the GMD and GSD at the engine exit plane

S3 Comparison of system loss correction factors with and without PSD measurement

S4 Average nvPM density calculation

S5 Measurement uncertainty estimation

S6 nvPM GMD_{EEP} and GSD_{EEP} as a function thrust and engine type

S1 PSD measurements with and without a catalytic stripper

Catalytic Stripper (CS) removes volatile and semi-volatile gas and particle phase in the sample through oxidation. A CS consists of a ceramic catalyst with metal coatings typically kept at 350°C operating temperature and a heat exchanger downstream of the catalyst to cool down the sample to <~50°C.

The hot exhaust sampling at the engine exit plane, heated sample lines and rapid dilution suppress the formation of volatile PM in ARP6320 systems. Extensive nvPM emission test campaigns with ARP6320 systems equipped with sizing instruments attached to Splitter 2 (Figure S1) did not find evidence of volatile PM at the instrument level (either a nucleation mode or a coarse mode) in tests when the system was operated as specified without any contamination.

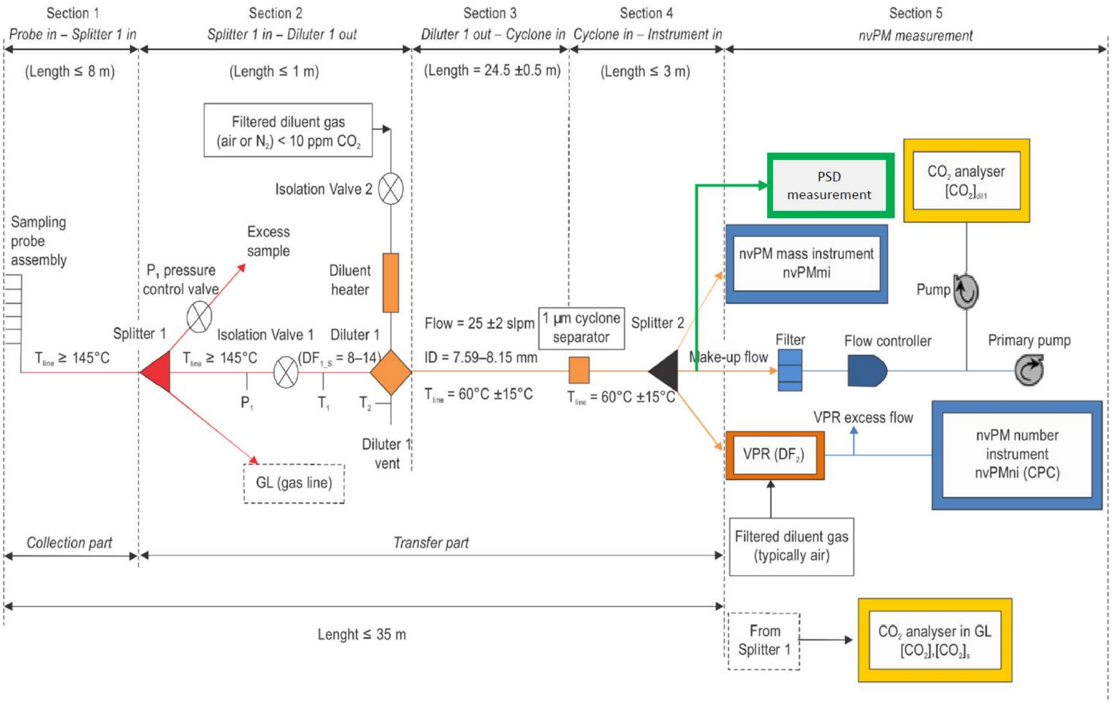

Figure S1 Schematic of an SAE ARP6320 / ICAO Annex 16 vol. II compliant nvPM sampling and measurement system with PSD measurement. Figure adapted from the ICAO Annex 16 vol. II, App. 7[1].

A CS (Models CS08 and CS015, Catalytic Instruments, GmbH) was used in the Swiss nvPM system during several engine tests upstream of the SMPS. The CS causes additional particle losses due to thermophoresis and diffusion that must be corrected. The manufacturer provides an empirically determined penetration function. This penetration function was verified experimentally and adjusted as it overpredicted losses of particles <~30 nm. Figure S2 shows PSDs from an engine with a rated thrust (F_{oo}) of ~120 kN measured with and without the CS. The samples with and without CS (bypassed) were measured consecutively at a stable engine condition. The distributions are averages of two consecutive scans. Note the different scales on the y-axes. No volatile PM was observed. The differences between the distributions shown are due to the random variability and sampling and measurement uncertainty.

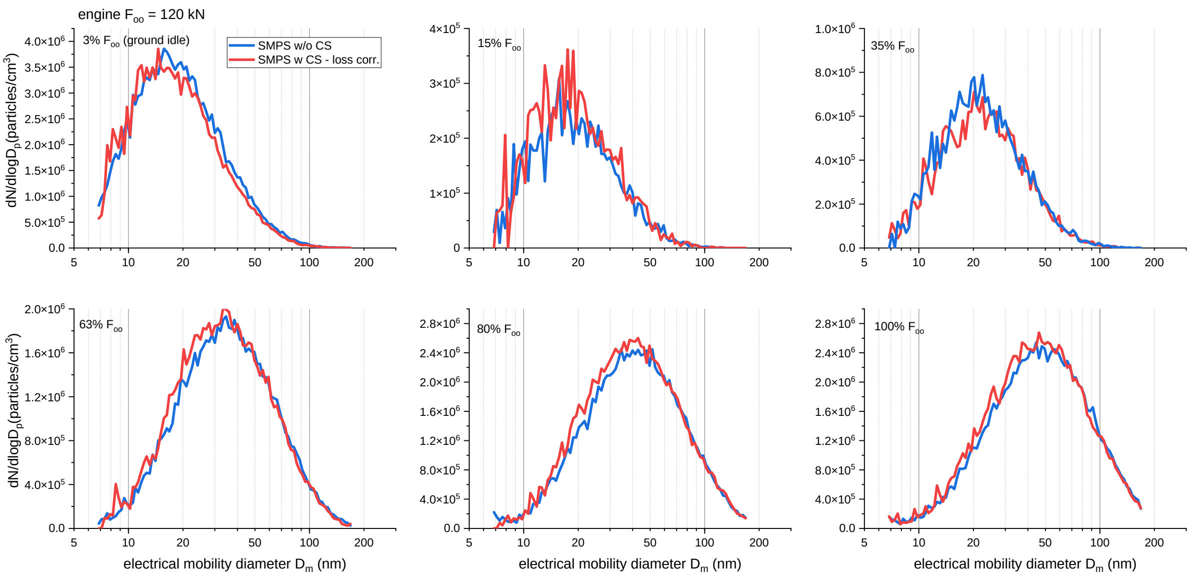

Figure S2 Example of PSD measurement without CS (blue line) and with CS (red line) at thrust levels from idle to takeoff (100% rated thrust F_{oo}).

Another example of an engine included in this study is a small turbofan engine with a mixed exhaust nozzle with a rated thrust ~16 kN (Figure S3)[2]. This engine was tested with regular Jet A-1 fuel without synthetic blending components and a 30% HEFA-SPK (hydrotreated esters and fatty acids – synthetic paraffinic kerosene) sustainable aviation fuel (SAF) blend. The PSD

was measured with a CS during one engine run and with a CS in the following run, i.e. no switching / bypassing of the CS was done during the test. No volatile PM was found in this test.

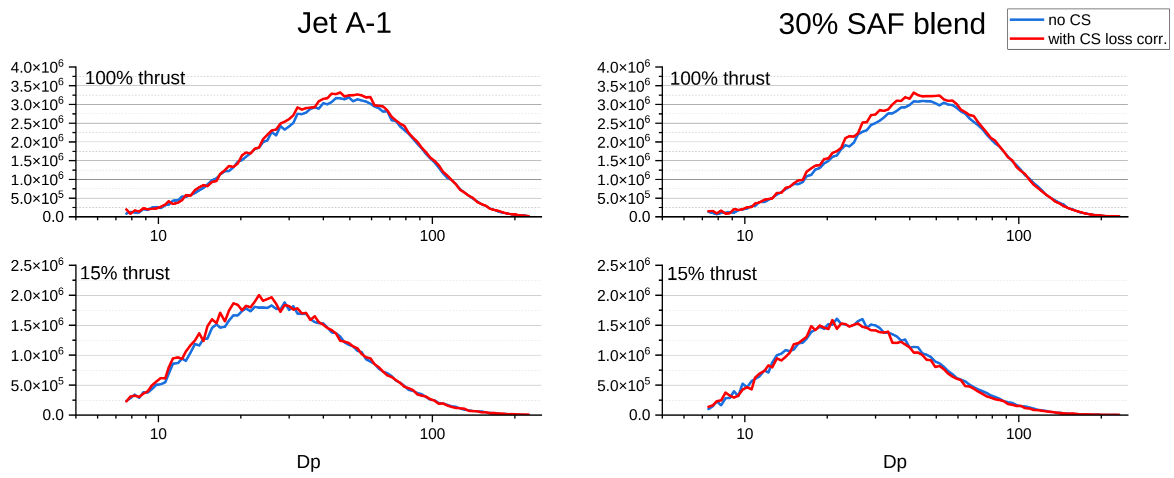

Figure S3 Example of PSD measurement without (blue line) and with CS (red line) for a mixed-exhaust small turbofan engine (rated thrust ~16 kN) at low thrust and at takeoff thrust burning regular Jet A-1 and a Jet A-1 containing 30% SAF.

The PSD measurements done without and with a CS (after loss correction) have shown no differences beyond random variability. All PSDs used in this study have not indicated any volatile PM contribution in the form of a secondary mode or significant volatile PM mass.

S2 Determination of the GMD and GSD at the engine exit plane

The lognormal GMD and GSD at the EEP were derived by fitting/minimizing the product of a lognormal distribution and penetration efficiency between the EEP and the instrument against the measured PSD (Figure S4). This method provides better convergence and reproducibility than fitting a lognormal distribution to loss-corrected size bins (open symbols) and it reduces the uncertainty <10 nm caused by high uncertainty in the PSD measurement and penetration function at those small sizes[3]. A consequence of these high uncertainties is a fictional secondary mode <10 nm that may occur in the loss-corrected size bins (Figure S4c, d). Moreover, fitting a lognormal PSD to loss-corrected size bins will not converge for GMD_{EEP} close to the lower size cutoff of the analyzer. The CH system data were processed using OriginPro software using

a fitting procedure. The EU system's DMS500 data were processed using a Matlab minimization routine. The Matlab routine could not be automatized for the CH system data due to the variable size range of the SMPS data and filling the missing size bins with zeroes led to false results. Using the same input, the two methods reported identical results.

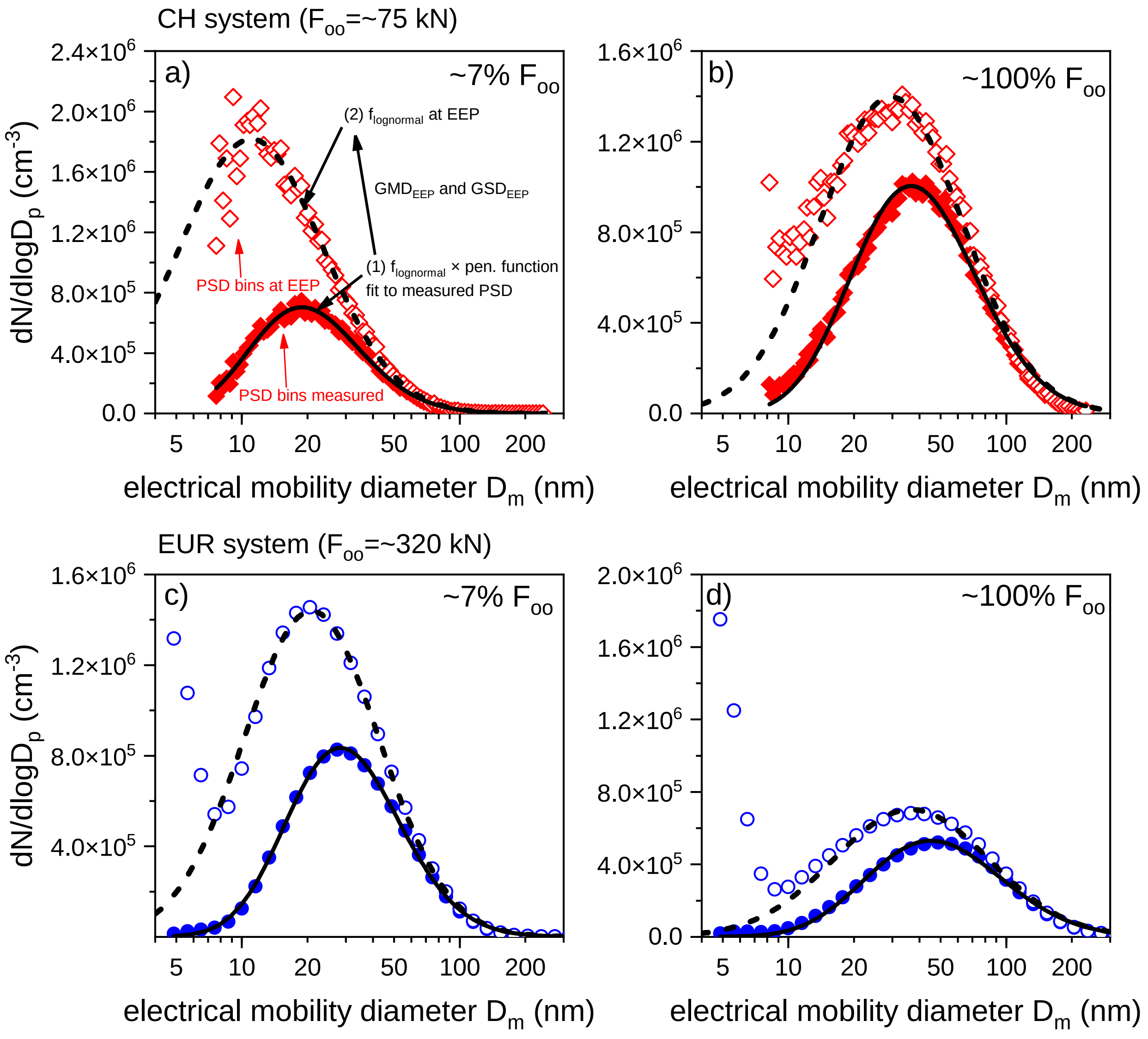

Figure S4 Visualization of the methodology for determination of the lognormal GMD and GSD at the EEP at idle and take-off for two engines measured using the CH system (a, b) and the EUR system (c, d). The solid curves represent the product of lognormal distribution and the system penetration function fitted to the measured PSDs. The dashed

curves represent the lognormal distributions modeled using the GMD and GSD obtained from the fitting procedure.

S3 Comparison of system loss correction factor with and without PSD measurement

Figure S5 shows a comparison of the system loss correction factors (k_{SL}) determined using the regulatory method (used in this study in line with reported standardized emissions certification data) and the newly proposed methodology utilizing PSD measurement[3]. The lower size cutoff used in both methods is 10 nm. The scatter increased with increasing N/M (decreasing GMD and nvPM mass concentration). The trend for k_{SLmass} is above the 1:1 line, suggesting that the assumed GSD of 1.8 in the regulatory method is underestimated (an average value of 2.05 was found in this study). However, GSD adjustment does not reduce the scatter, as demonstrated previously[3]. While the differences in the k_{SL} were up to 70% (i.e. a factor of 1.7 overestimation of the regulatory k_{SL} compared to PSD-based k_{SL}), the ratio of the correction factors applied to the N/M measured (i.e. k_{SLnum}/k_{SLmass}) using the regulatory method and the PSD-based method ranged from 0.8 to 1.25 (mean 0.97). In other words, the trends reported as a function of N/M_{SL} in the main paper would not significantly differ when the PSD-based k_{SL} are applied.

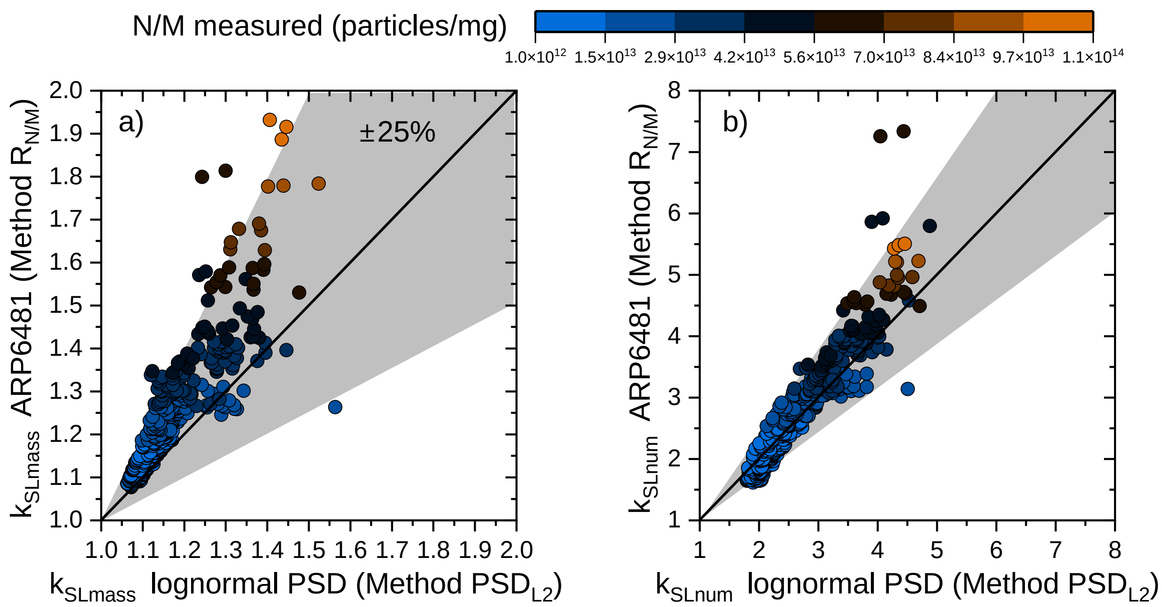

Figure S5 Comparison of the system loss correction factors (k_{SL}) for nvPM mass (a) and nvPM number (b) determined using the regulatory method without PSD measurement (method $R_{N/M}$) and with PSD measurement (method PSD_{L2}). The nomenclature and details are explained in Durand et al. 2023[3].

S4 Average nvPM density calculation

The nvPM emission tests in this study have shown inconsistencies between the number concentrations reported by the nvPM number instrument and the PSD instrument over the years. While the data is strongly correlated (R^2>0.95), the slope varied by ±25% from the 1:1 line. A comparison of DMS500 total number and APC nvPM number for four engines included in this study is shown in Durand (2019)[4]. A similar degree of variability has been observed for the SMPS-reported total number concentration with respect to the APC nvPM number. The differences were not engine specific. The relative agreement between the instruments varied between instrument calibrations and test campaigns of the same engine types. Since the APC number concentrations reported by the EUR and CH systems have been shown to agree well with each other with no observable drift [5,6], we corrected the PSD-based total number concentrations to APC nvPM number.

In this correction, first, the PSDs reported by the DMS500 and SMPS were corrected to the measurement plane of the CPC in the APC (Figure S1). The concentrations in each size bin were corrected for size-dependent losses in the APC VPR and for the CPC cutoff. With the integrated PSD number and APC number now reported at the same location, the ratio of these two values was used to correct the integrated PM volume reported by the PSD instrument. The average nvPM density is then the nvPM mass reported by the MSS divided by the corrected integrated PSD volume.

Figure S6a shows a comparison of the average nvPM density calculated using the number concentrations reported by the size instruments (open circles) and the concentrations corrected to the APC number (red circles). The APC-corrected density was on average ~10% higher, (0.74 g/cm^3) than the one obtained using the PSD-based number concentration (0.67 g/cm^3). Apart from the reduced scatter, the trends and magnitude of the average densities remained the same. Interestingly, the data indicates two potential trend lines, which are likely a function of engine/combustor type. These trends can be observed for both systems (Figure S6b).

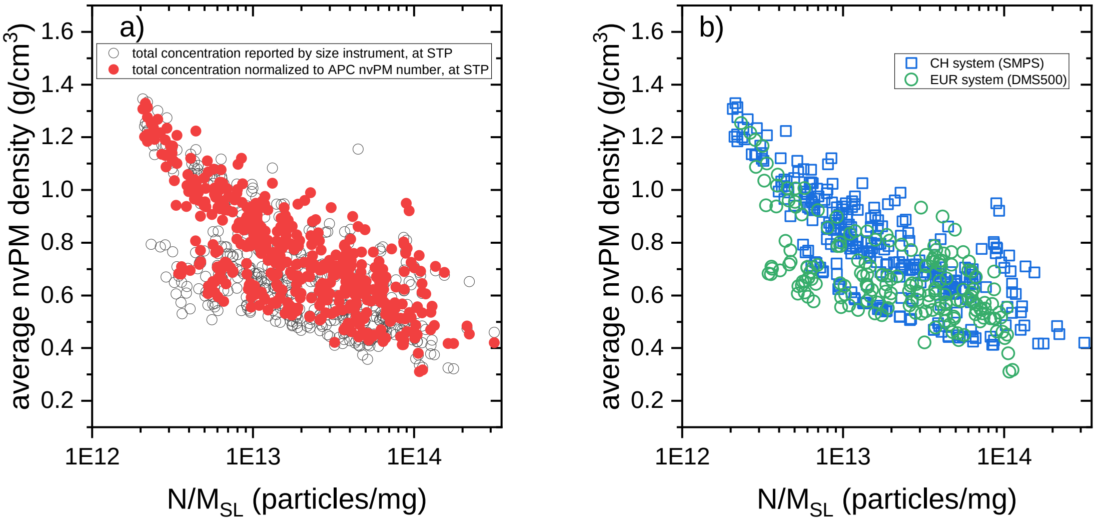

Figure S6 Comparison of average nvPM densities calculated using the total concentration reported by the size instrument and the nvPM number instrument (a). The latter is shown in panel (b), differentiated by the size instrument used.

S5 Measurement uncertainty estimation

The measurement uncertainties are estimated using typical figures provided in the standardized methods and literature[3,4,7]. The nvPM mass and number measurements have an estimated total uncertainty (95% confidence interval) of 19.7% (propagated 10% uncertainty of the measurement and 16.7% of the calibration) and 14.1% (propagated 10% uncertainty of the particle counter and 10% of the dilution factor (DF2, Figure S1)), respectively. These figures may be underestimated, especially at low nvPM mass concentrations close to the limit of detection and particle sizes <~20 nm [4].

The penetration function uncertainty in the regulatory method is a function of particle size, with a maximum estimated value of 12%. The uncertainties in the regulatory system loss correction factors were previously modeled using the Monte Carlo Method[7]. The uncertainties in the system loss correction factors increase with decreasing GMD. At 10 nm GMD, the number and mass-based correction factors have estimated uncertainties of 60% and 25%. At 40 nm, the uncertainty decreases to 45% for the number correction factor and 3% for the mass correction factor. Thus, the propagated uncertainty at 95% confidence of the N/M at the engine exit plane is ~69% for GMD = 10 nm and ~51% for GMD = 40 nm. These uncertainties do not include potential additional uncertainties due to measurements at the limit of detection or instrument calibration drift.

In the average nvPM density calculation, we consider the sizing uncertainty (5% for GMD and GSD based on intercomparisons of the SMPS and DMS500[3]) of the fitted lognormal distributions, number concentration uncertainty (20%, reported DMS500 number uncertainty; the uncertainty due to correction to APC concentration is not considered separately) and the nvPM mass uncertainty (19.7%).

The average nvPM density is calculated as:

$$\rho = \frac{nvPMmass}{V_{total}}$$

The integrated PSD volume V_{total} can be expressed as:

$$V_{total} = \int_{1\ nm}^{1000\ nm} \left(\frac{N}{(2\pi)^{0.5} D_p \ln GSD} \exp(-\frac{(\ln D_p - \ln GMD)^2}{2\ln^2 GSD} \frac{4}{3}\pi \left(\frac{D_p}{2}\right)^3 \right) dD_p$$

The relative uncertainty for V_{total} can be expressed as:

$$\frac{\Delta V_{total}}{V_{total}} \approx \sqrt{\left(\frac{\partial V_{total}}{\partial N}\frac{\Delta N}{V_{total}}\right)^2 + \left(\frac{\partial V_{total}}{\partial GMD}\frac{\Delta GMD}{V_{total}}\right)^2 + \left(\frac{\partial V_{total}}{\partial GSD}\frac{\Delta GSD}{V_{total}}\right)^2}$$

Each partial derivative in the uncertainty propagation equation represents the sensitivity of the total volume to changes in the respective variable. The estimate for the relative uncertainty of the total volume of the lognormally distributed aerosol particles, given the uncertainties in the total number concentration, geometric mean diameter, and geometric standard deviation, is ~40%.

The relative uncertainty in the density $\frac{\Delta\rho}{\rho}$ can be calculated using the following equation:

$$\left(\frac{\Delta\rho}{\rho}\right)^2 = \left(\frac{\Delta nvPMmass}{nvPMmass}\right)^2 + \left(\frac{\Delta V_{total}}{V_{total}}\right)^2$$

The estimated propagated uncertainty is ~45%.

S6 nvPM GMD_{EEP} and GSD_{EEP} as a function of thrust and engine type

In Figure 1 of the main paper, we present GMD_{EEP} and GSD_{EEP} as a function of engine thrust for the combined dataset of a wide range of engine types. The thrust dependence of GMD_{EEP} and GSD_{EEP} for each of the 19 engine types is shown in Figures S7-S9 below. Some figures include measurements of multiple engines (n>1).

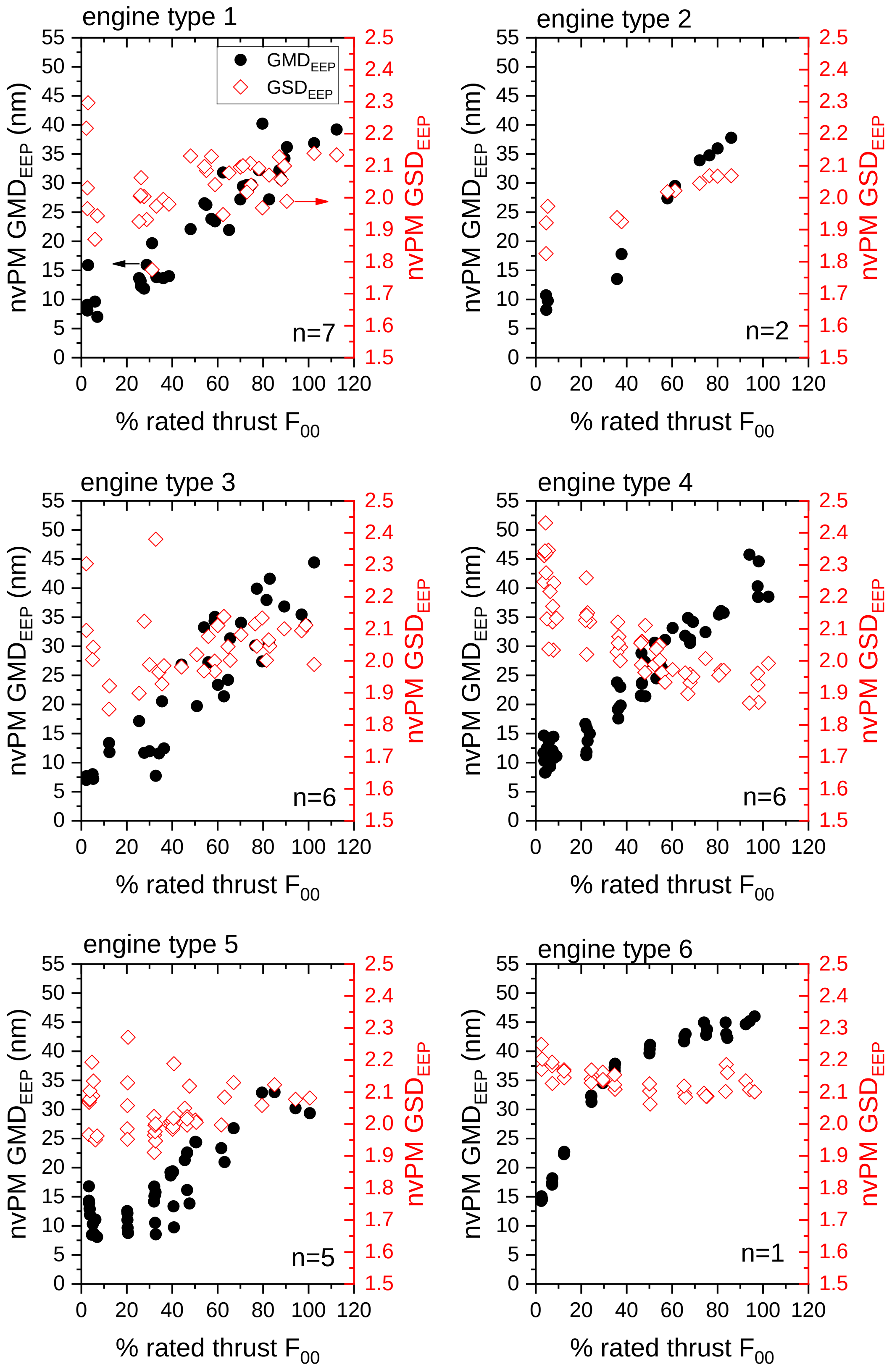

Figure S7 nvPM GMD (left y-scale) and GSD (right y-scale) at the engine exit plane for as a function of thrust for engine types 1-6.

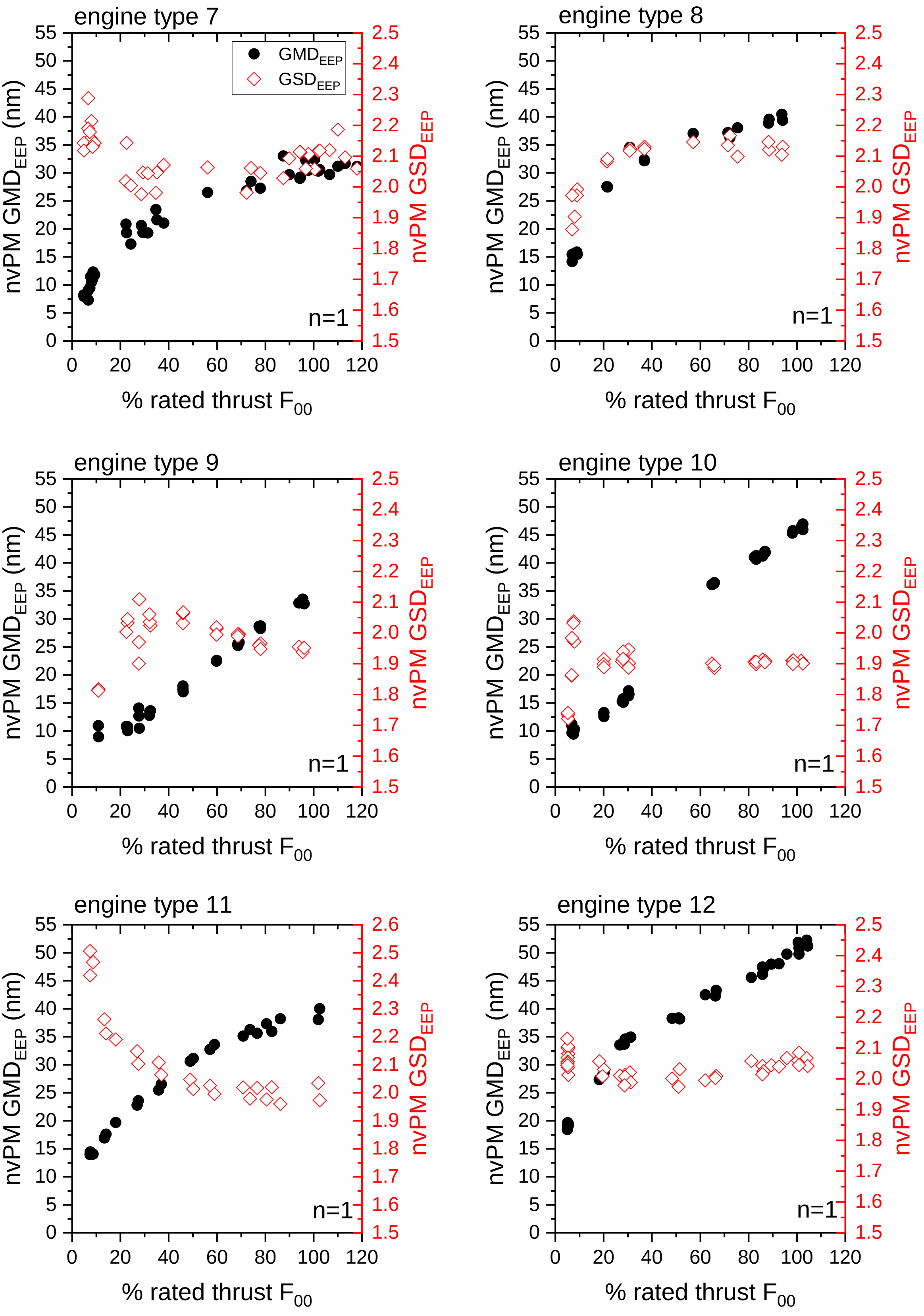

Figure S8 nvPM GMD (left y-scale) and GSD (right y-scale) at the engine exit plane for as a function of thrust for engine types 7-12.

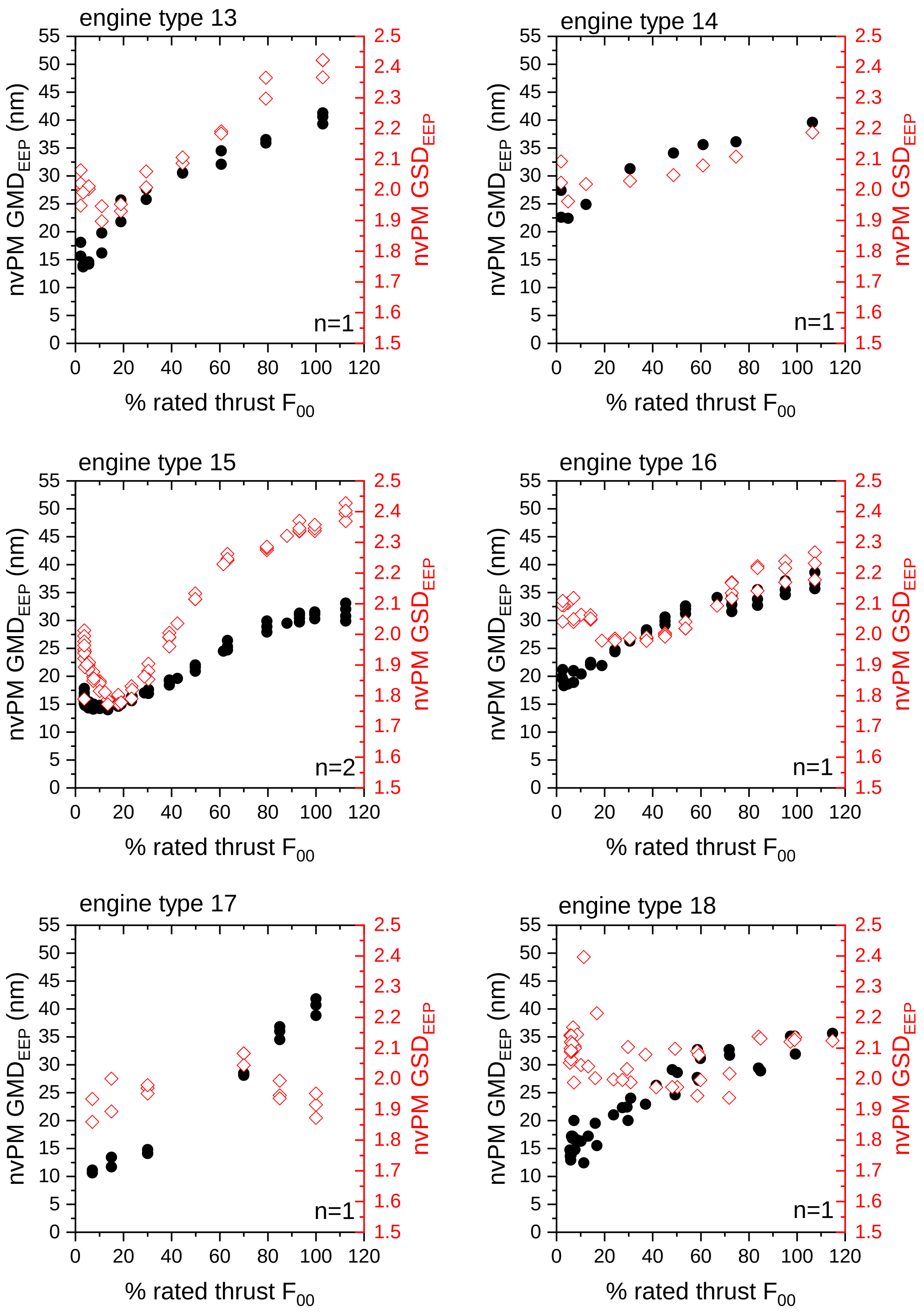

Figure S9 nvPM GMD (left y-scale) and GSD (right y-scale) at the engine exit plane for as a function of thrust for engine types 13-18.

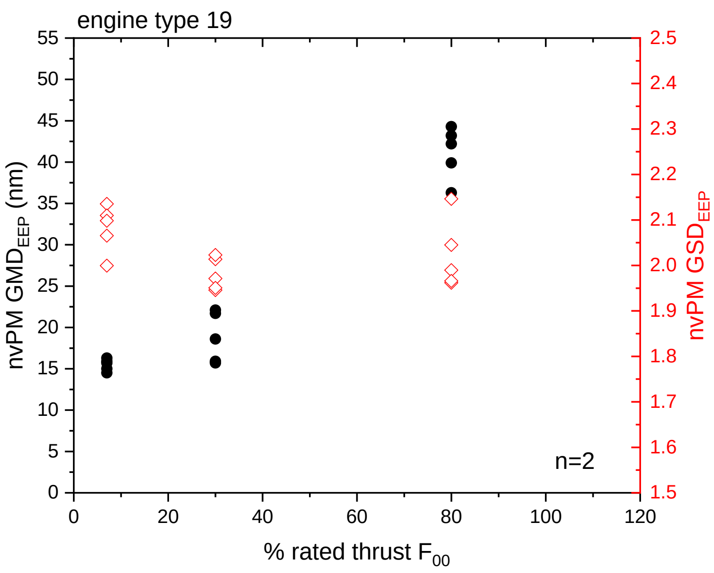

Figure S10 nvPM GMD (left y-scale) and GSD (right y-scale) at the engine exit plane for as a function of thrust for engine type 19.

References

(1) ICAO. *Annex 16 - Environmental Protection Volume 2 - Aircraft Engine Emissions*; 2017. https://store.icao.int/annex-16-environmental-protection-volume-2-aircraft-engine-emissions.html (accessed 2019-02-18).

(2) Durdina, L. Swiss Research of Particle Emissions Reduction with Sustainable Aviation Fuels, 2023. https://nanoparticles.ch/archive/2023_Durdina_PR.pdf (accessed 2024-02-19).

(3) Durand, E.; Durdina, L.; Smallwood, G.; Johnson, M.; Spirig, C.; Edebeli, J.; Roth, M.; Brem, B.; Sevcenco, Y.; Crayford, A. Correction for Particle Loss in a Regulatory Aviation nvPM Emissions System Using Measured Particle Size. *J. Aerosol Sci.* **2023**, *169*, 106140. https://doi.org/10.1016/j.jaerosci.2023.106140.

(4) Durand, E. F. Towards Improved Correction Methodology for Regulatory Aircraft Engine nvPM Measurement, Cardiff University, 2019. https://orca.cardiff.ac.uk/id/eprint/126400/1/2019DurandEFPhD.pdf (accessed 2024-02-29).

(5) Lobo, P.; Durdina, L.; Brem, B. T.; Crayford, A. P.; Johnson, M. P.; Smallwood, G. J.; Siegerist, F.; Williams, P. I.; Black, E. A.; Llamedo, A.; Thomson, K. A.; Trueblood, M. B.; Yu, Z.; Hagen, D. E.; Whitefield, P. D.; Miake-Lye, R. C.; Rindlisbacher, T. Comparison of Standardized Sampling and Measurement Reference Systems for Aircraft Engine Non-Volatile Particulate Matter Emissions. *J. Aerosol Sci.* **2020**, 105557. https://doi.org/10.1016/j.jaerosci.2020.105557.

(6) Crayford, A.; Durand, E.; Delhaye, D.; Durdina, L.; Ortega, I. K.; Williams, P. *RAPTOR Work Package 4: PM Measurements Deliverables Report*; 2022. https://zenodo.org/records/7385796 (accessed 2022-10-07).

(7) SAE International. ARP 6481- Procedure for the Calculation of Sampling Line Penetration Functions and Line Loss Correction Factors. **2019**. https://doi.org/10.4271/ARP6481.

(8) ISO. *Determination of Particle Size Distribution. Differential Electrical Mobility Analysis for Aerosol Particles*, Definitive.; 2020.
